# Discovery of VU6066098: A Selective and CNS-Penetrant mGlu_2_ NAM with Robust Antidepressant‑, Antipsychotic‑, and Procognitive-like Activity in Rodents

**DOI:** 10.1021/acschemneuro.6c00324

**Published:** 2026-06-23

**Authors:** Jeremy S. Coleman, Rory A. Capstick, Sichen Chang, Derek J. Rios, Michael Bubser, Analisa D. Thompson Gray, Irene Zagol-Ikapitte, Srinivasan Krishnan, Hyekyung P. Cho, Alice L. Rodriguez, Colleen M. Niswender, Olivier Boutaud, Georgina Perez Garcia, Gregory A. Elder, Darren W. Engers, Carrie K. Jones, Craig W. Lindsley

**Affiliations:** † Warren Center for Neuroscience Drug Discovery, 12328 Vanderbilt University, Nashville, Tennessee 37232, United States; ‡ Department of Pharmacology, Vanderbilt University School of Medicine, Nashville, Tennessee 37232, United States; § Department of Chemistry, Vanderbilt University, Nashville Tennessee 37232, United States; ∥ Vanderbilt Institute of Chemical Biology, Vanderbilt University, Nashville, Tennessee 37232, United States; ⊥ Vanderbilt Institute for Therapeutic Advances, Vanderbilt University, Nashville, Tennessee 37232, United States; # Research and Development Service, James J. Peters Department of Veterans Affairs Medical Center, 130 West Kingsbridge Road, Bronx, New York, New York 10468, United States; ∇ Department of Neurology, Icahn School of Medicine at Mount Sinai, One Gustave Levy Place, New York, New York 10029, United States; ○ Department of Psychiatry, Icahn School of Medicine at Mount Sinai, One Gustave Levy Place, New York, New York 10029, United States; ◆ Mount Sinai Alzheimer’s Disease Research Center and Ronald M. Loeb Center for Alzheimer’s Disease, Icahn School of Medicine at Mount Sinai, One Gustave Levy Place, New York, New York 10029, United States; ¶ Neurology Service, James J. Peters Department of Veterans Affairs Medical Center, 130 West Kingsbridge Road, Bronx, New York, New York 10468, United States; ⧅ Vanderbilt Brain Institute, Vanderbilt University, Nashville, Tennessee 37240, United States; †† Vanderbilt Kennedy Center, Vanderbilt University, Nashville, Tennessee 37203, United States

**Keywords:** metabotropic glutamate receptor subtype 2 (mGlu_2_), novel object recognition (NOR), amphetamine-induced hyperlocomotion (AHL), contextual fear conditioning (CFC), blast-related traumatic brain injury (TBI), forced swim, depression, negative allosteric modulator (NAM)

## Abstract

Herein, we report the discovery and development of an optimized mGlu_2_ Negative Allosteric Modulator (NAM) *in vivo* tool compound, VU6066098, based on a novel, structurally distinct chemotype. VU6066098 is a potent, selective, and CNS-penetrant mGlu_2_ NAM with excellent rat PK (CL_p_ = 23.9 mL/min/kg, *t*_1/2_ = 2.2 h, %F = 100, K_p_ = 1.28, K_p,uu_ = 0.25), making it ideal to explore the therapeutic potential of selective mGlu_2_ inhibition in preclinical rat models. In a rat forced swim test, VU6066098 displayed an oral minimum effective dose (MED) of 1 mg/kg and was equi-efficacious to ketamine. In amphetamine-induced hyperlocomotion, VU6066098 displayed an oral minimum effective dose (MED) of 30 mg/kg. While in the preclinical cognitive tasks of rat novel object recognition and acquisition of contextual fear conditioning, VU6066098 produced robust dose-dependent effects at oral minimum effective doses (MED) of 3 mg/kg and 0.3 mg/kg, respectively. In a blast-related traumatic brain injury (TBI) model, administration of VU6066098 at a dose of 10 mg/kg IP was effective acutely, and the effect on NOR memory was sustained up to 30 days postdose. Thus, mGlu_2_ NAMs show therapeutic potential for the treatment of a broad range of affective and cognitive symptoms associated with Major Depressive Disorder, Alzheimer’s disease, TBI, and acute psychosis; moreover, these data strongly support further optimization of mGlu_2_ NAMs for future clinical development.

## Graphical abstract

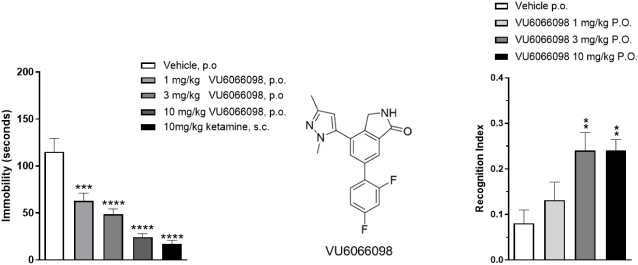

## Introduction

Among the eight members (mGlu_1–8_) of the metabotropic glutamate receptor family, presynaptic group II metabotropic glutamate receptors (mGlu_2_ and mGlu_3_) still lack *in vivo* tool compounds to assess the therapeutic potential of selective mGlu_2_ inhibition and mGlu_3_ activation. Both mGlu_2_ and mGlu_3_ are widely expressed in the mammalian CNS within neural circuitry known to be disrupted in many CNS disorders (e.g., anxiety, Major Depressive Disorder (MDD), schizophrenia, chronic pain, Substance Use Disorders, Alzheimer’s disease (AD), and Parkinson’s disease (PD)).^1–8^ Early *in vitro* and *in vivo* pharmacology studies were reported using dual orthosteric mGlu_2/3_ antagonists, agonists, or dual mGlu_2/3_ negative and positive allosteric modulators (NAMs and PAMs).^9–14^ While discrete roles for each subtype remained unclear from these studies, it was apparent that, for many CNS indications, dual modulation of mGlu_2_ and mGlu_3_ was not ideal, and that subtype-selective *in vivo* tools were required. Recently, highly selective mGlu_3_ NAMs and mGlu_2_ PAMs have been reported with DMPK properties suitable for *in vivo* dosing.^15–20^ A highly selective orthosteric mGlu_3_ agonist has been reported by Lilly,^21^ but no mGlu_3_ PAMs have yet to be disclosed. There have been advances in the development of mGlu_2_ NAMs (Figure 1) and are represented by **2**–**6** (as well as a clinical mGlu_2/3_ dual NAM, **1**).^22–25^ First-generation mGlu_2_ NAM ligands (e.g., **2**,**4–6**) are characterized by poor physicochemical and DMPK properties (e.g., high lipophilicity, low *f*_u_, and poor solubility), high plasma clearance (CL_p_) and short half-life (*t*_1/2_), low CNS penetration (rat brain:plasma partition ratios, or *K*_p_s of ≤0.3) and low oral bioavailability (<50%). Even the Merck clinical candidate **3** showed modest rat PK (CL_p_ = 36 mL/min/kg (>50% Q_h_), and modest oral bioavailability (32%F). All of the mGlu_2_ NAMs reported harbor a conserved primary carboxamide as a requisite pharmacophore, which is also a DMPK liability, leading to poor-to-moderate CNS exposure and moderate PK (e.g., conversion to the corresponding carboxylic acid). Thus, we felt the field needed an improved *in vivo* tool to fully explore the therapeutic potential of mGlu_2_ NAMs, and this would require the development of a new chemotype (devoid of the primary carboxamide moiety) and diverse *in vivo* profiling across multiple pharmacodynamic assays.

**Figure 1:**
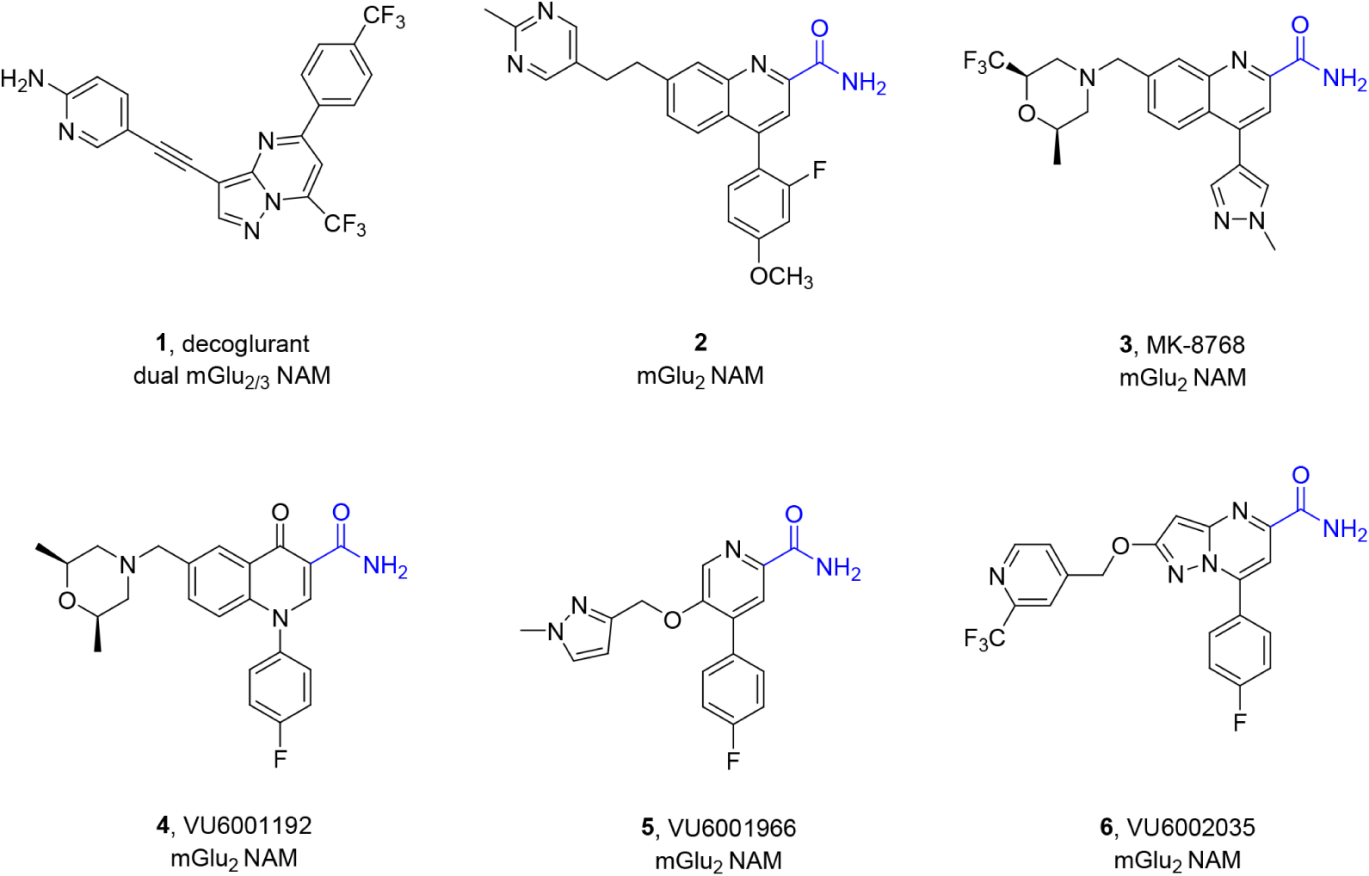
Structures of decoglurant (**1**), an mGlu_2/3_ dual NAM, and **2**-**6**, selective mGlu_2_ NAMs that possess a key primary carboxamide pharmacophore.

## Results and Discussion

### Design

As discussed, the main driver of design was to replace the primary carboxamide of a prototypical mGlu_2_ NAM **5**, as well as replace other potentially labile moieties such as the ether linkage in the 5-position (Figure 2). Toward these goals, we questioned if cyclization of the primary carboxamide onto the central arene core to afford a stable lactam congener would also maintain mGlu_2_ NAM activity. In parallel, we hypothesized that deletion of the ether linkage to the pyrazole would rigidify the compound and replace yet another metabolic liability, as well as a hydrogen bond donor, to improve CNS penetration. While contrary to all historical SAR data wherein the primary carboxamide is an essential pharmacophore, this approach proved successful. Moreover, SAR on the southern phenyl moiety was limited to either 4- or 2,4-disubstitution, and, in the present case, a 2,4-difluorophenyl group proved optimal. Here, we highlight the culmination of this effort with VU6066098 (**7**), a potent (human IC_50_ = 77 nM) and selective mGlu_2_ NAM (mGlu_3_ IC_50_ > 30 μM) with excellent rat CNS penetration (K_p_ = 1.28) and PK (CL_p_ = 23.9 mL/min/kg, *t*_1/2_ = 2.2 h, %F = 100) to allow *in vivo* POC studies to be performed.

**Figure 2:**
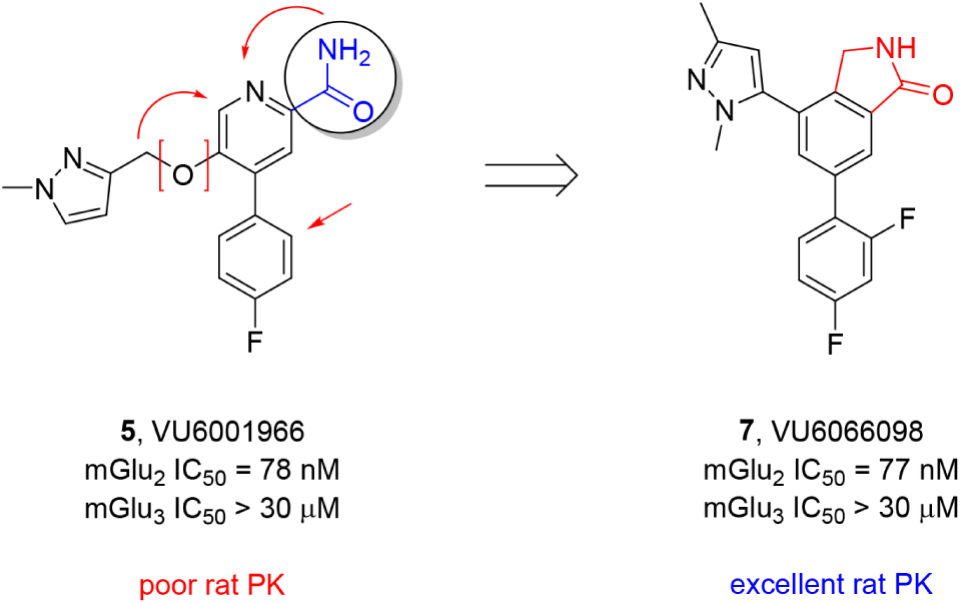
Optimization of NAM **5** to afford NAM **7**. Cyclization of the primary carboxamide onto the core ring affords a stable lactam. Removal of the ether linkage and direct C–C bind between the core aromatic ring and the pyrazole further removes metabolic liabilities and another hydrogen bond acceptor.

### Synthesis

A high-yielding, four-step route for the multigram synthesis of NAM **7** was developed (Scheme 1). Starting from commercial ester **8**, benzylic bromination to deliver **9** proceeded in 85% yield. Aminolysis of the methyl ester and concomitant S_N_2 displacement of the benzylic bromide provided the desired lactam **10** in 83% yield. Next, a chemoselective Suzuki coupling with the aryl bromide under microwave-assisted conditions gave biaryl **11** in 99% yield. Finally, a “hotter” palladium catalyst (5 mol % RuPhos-G3-palladacycle) facilitated a Suzuki coupling with the aryl chloride to install the pyrazole, delivering **7** (VU6066098) in 78% yield. With gram quantities of NAM **7** in hand, the full *in vitro* and *in vivo* profiling could commence.

**Scheme 1:**
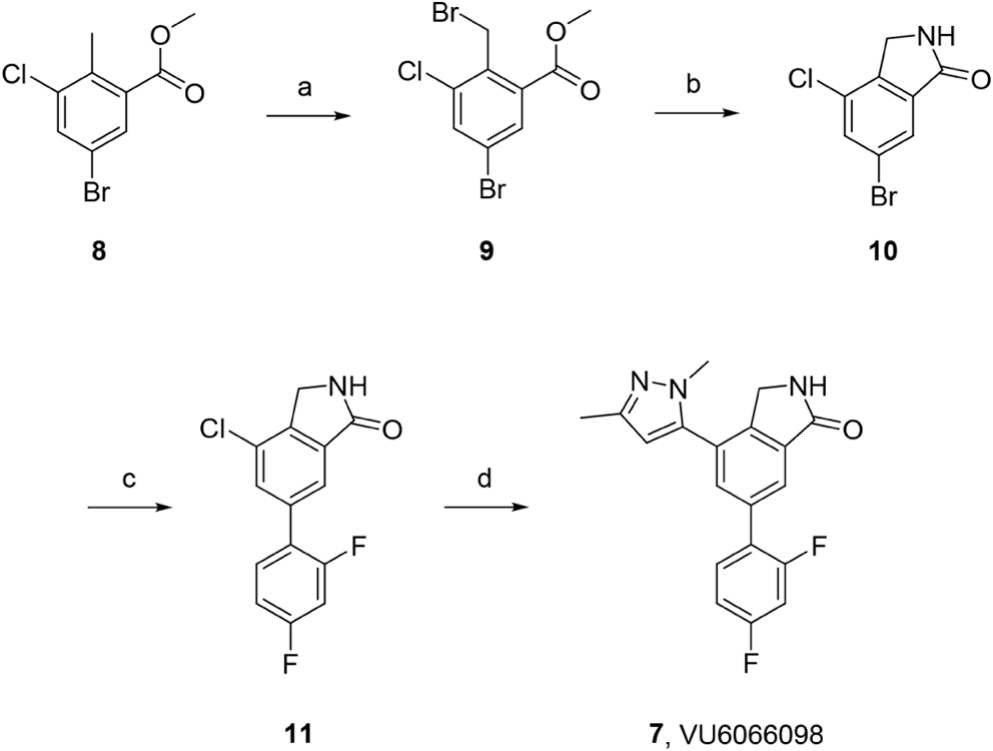
Synthesis of VU6066098 (**7**)­^a^

a Reagents and conditions: (a) NBS, AIBN, CCl_4_, 90 °C, 3 h, 85% (b) NH_3_, MeOH, 100 °C, 3 h, 83% (c) 2,4-difluorophenyl boronic acid, 5 mol % Pd­(dppf)­Cl_2_·DCM, CsCO_3_, 1,4-dioxane:H_2_O (5:1), 120 °C, μw, 15 min, 99% (d) (1,3-dimethyl-1*H*-pyrazol-5-yl)­boronic acid, K_2_CO_3_, 5 mol % RuPhos-G3-palladacycle, THF/H_2_O (3:1), 140 °C, μw, 15 min, 78%.

### Molecular Pharmacology and DMPK

NAM **7** was an attractive small molecule tool from a physicochemical, pharmacological and DMPK perspective. To begin with, NAM **7** possessed acceptable solubility under both neutral (pH 6.8 = 10.3 μM) and acidic (pH 2.2 = 34.1 μM) conditions (though these would need to be improved for a clinical candidate). NAM **7** was low molecular weight (339) with a favorable xLogP (3.9) for a GPCR allosteric modulator (i.e., binding site in lipophilic transmembrane domains), and Lipinski compliant. Novel NAM **7** was equipotent (human mGlu_2_ IC_50_ = 77 nM) to the prototypical carboxamide **5** (human mGlu_2_ IC_50_ = 78 nM) in our mGlu_2_/GIRK assay and was inactive (>10 μM) on human mGlu_1,3,4,5,7_ and a weak PAM of human mGlu_8_ (EC_50_ = 5.2 μM, 49% Glu Max). The activity of NAM **7** was confirmed in a human cAMP assay (mGlu_2_ IC_50_ = 114 nM). In rat cell lines, NAM **7** displayed comparable activity (rat mGlu_2_ IC_50_ = 111 nM) and selectivity (inactive (>10 μM) on rat mGlu_1,3,4,5,7_ and a weak PAM of rat mGlu_8_ (EC_50_ = 857 nM, 59% Glu Max)). In the CEREP safety screen of 44 targets, NAM **7** was clean (no inhibition >50% @10 μM), indicating its utility as a quality tool compound.

The *in vitro* DMPK profile of NAM **7** was also attractive. Good unbound fraction was observed in human plasma (*f*_u_ = 0.027), rat plasma (*f*_u_ = 0.070) and mouse plasma (*f*_u_ = 0.128) as well as in brain homogenate binding (rat brain (*f*_u_ = 0.013) and mouse brain (*f*_u_ = 0.019)). Moderate predicted hepatic clearance was seen for human (CL_hep_ = 11.1 mL/min/kg) and rat (CL_hep_ = 36.6 mL/min/kg), while mouse clearance was high (CL_hep_ = 73.9 mL/min/kg), suggesting a need for profiling of *in vivo* activity in rat preclinical models. In a rat IV/PO PK study, NAM **7** showed low clearance (CL_p_ = 12.7 mL/min/kg) with a reasonable half-life (*t*_1/2_ = 2.4 h) and excellent oral bioavailability (100%F). In a rat plasma:brain level (PBL) study, **7** had a high K_p_ of 1.28 and a K_p,uu_ of 0.25 (due to higher rat BHB than plasma PPB). Moreover, NAM **7** was not likely a human P-gp substrate (MDCK-MDR1 ER = 3.5) and good P_app_ (15.9 × 10^–6^ cm/s). Thus, NAM **7** overcame many of the limitations of earlier mGlu_2_ NAMs and was advanced into pharmacodynamic rat models to validate selective mGlu_2_ inhibition.

### Pharmacodynamic Rat Models

With the optimization of this selective, highly CNS-penetrant mGlu_2_ NAM, we assessed the efficacy of this ligand across multiple pharmacodynamic rat models of symptoms observed in various neuropsychiatric and neurologic disorders to inform the therapeutic potential of selective mGlu_2_ inhibition.

### Antidepressant-like Activity

There is a long history with dual mGlu_2/3_ NAMs demonstrating efficacy in preclinical models of antidepressant-like activity comparable to ketamine;^26,27^ however, in human clinical trials, the dual mGlu_2/3_ NAM decoglurant (**1**) failed to exert antidepressant or procognitive effects, despite being well tolerated.^28^ While the lack of procognitive efficacy with a dual NAM was not unexpected, the lack of antidepressant-like activity was surprising. Particularly since Joffe et al. had previously demonstrated that both mGlu_2_ and mGlu_3_ selective NAMs divergently enhance thalamocortical transmission and exert rapid antidepressant effects (e.g., latency to immobilize) in mice, although the tool compounds used were not optimal.^13^ Further support for mGlu_2_ inhibition was the finding that the active metabolite of ketamine, (2*R*,6*R*)-hydroxynorketamine, exerts mGlu_2_ receptor-dependent antidepressant actions, and the authors further indicated that combination therapy of ketamine with an mGlu_2_ inhibitor could be of therapeutic value.^29^ Thus, we evaluated NAM **7** in a rat forced swim test, a classic preclinical model predictive of antidepressant-like activity,^30^ in comparison with the effects of ketamine. In a dose-dependent manner, NAM **7** reduced immobility with a minimum effective dose (MED) of 1 mg/kg p.o., and at 10 mg/kg provided comparable efficacy to a 10 mg/kg s.c. dose of ketamine (Figure 3). Satellite PK sample analysis demonstrated that 230 nM total brain levels were achieved at the 1 mg/kg dose p.o. MED (2.1-fold the rat IC_50_) and 3.1 nM free brain levels (0.03-fold the rat IC_50_), suggesting that efficacy correlated with total, not free, brain concentrations. At the 10 mg/kg p.o. dose of NAM **7**, total brain concentrations achieved were 4.9 μM (43.9-fold the rat IC_50_) and 67 nM free brain concentrations (0.59-fold the rat IC_50_). Thus, selective inhibition of mGlu_2_ exerts rapid antidepressant-like effects comparable to ketamine.

**Figure 3:**
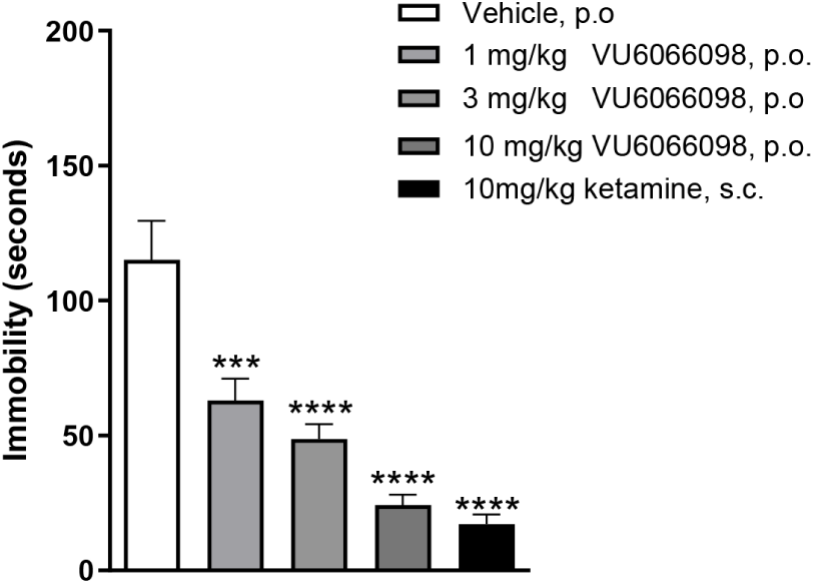
The NAM **7** produced a dose-dependent reversal of immobility time in the rat forced swim test, significant at doses of 1, 3, and 10 mg/kg p.o. compared to ketamine 10 mg/kg s.c. [F_4, 34_ = 24.02; ***p < 0.005 vs vehicle; ****p < 0.0001 vs vehicle (1-way ANOVA followed by Dunnett’s test)]. MED was 1 mg/kg p.o. with efficacy comparable to ketamine at 10 mg/kg s.c. Vehicle: 10% Tween 80 p.o. microsuspensions, *N* = 7–8/group.

### Antipsychotic-like Activity

In the field of novel treatment development for the symptoms associated with schizophrenia, most attention has been focused on mGlu_2/3_ agonists and mGlu_2_ PAMs for acute psychosis, while selective mGlu_3_ activation has been suggested to have procognitive functions.^31^ Due to the abundant distribution of mGlu_2_ in the basal ganglia and thalamostriatal projections into the striatum, mGlu_2_ plays a key role in learning, cognition and motor functions.^13^ It is fair to say that results thus far with genetic animal studies and pharmacological challenges with mGlu_2/3_ agonists and mGlu_2_ PAMs have been mixed.^31,32^ Recently, the mGlu_2_ PAM SAR218645 was shown to improve memory, reduce head twitch, but did not reverse hyperlocomotive activity induced by either amphetamine or MK-801.^33^ As the field had yet to evaluate a selective mGlu_2_ NAM in reversal of amphetamine-induced hyperlocomotion (AHL), a traditional preclinical model of antipsychotic-like activity,^34,35^ we evaluated NAM **7** in this paradigm. Under our standard AHL paradigm (Figure 4), rats were habituated for 30 min prior to the administration of either vehicle (10%Tween 80) or NAM **7**, followed 30 min later by a dose of amphetamine (0.75 mg/kg s.c.). Rats treated with vehicle/amphetamine displayed robust hyperactivity (peak activity ∼1600 beam breaks/5 min bin). Pretreatment with the NAM **7** reversed amphetamine-induced hyperlocomotion in a dose- dependent manner, significant at doses of 30 and 56.6 mg/kg p.o. Moreover, the magnitude of the effects observed with the top dose of the NAM 7 was comparable to the effects with the positive control M_4_ PAM VU0467154 (10 mg/kg p.o.) as well as other clinical available antipsychotic mechanisms. In comparison with the observed effects of NAM **7** when dosed alone in the preclinical models of antidepressant-like activity and cognitive enhancement, significantly higher doses of NAM 7 were required to reversed the amphetamine-induced changes in locomotor activity. In terms of PK/PD in this paradigm, the MED of NAM 7 was 30 mg/kg p.o., with compound levels of 12.9 μM total brain and 174 nM free brain (or 1.55-fold the rat IC_50_). At the top dose of 56.6 mg/kg p.o. of NAM **7**, total and free brain levels were 14.9 μM and 202 nM, respectively (or 1.79-fold the rat IC_50_). Thus, the robust PK/PD relationship in reversing rat AHL appeared to be driven by free brain concentrations, in accord with the M_4_ PAM (10 mg/kg p.o., total and free brain levels were 880 nM and 58.8 nM, respectively (or 1.66-fold the rat EC_50_). Based on these findings, it is possible that earlier generation mGlu_2_ NAMs could not reach unbound brain exposure at or above the rat IC_50_. While very exciting, an mGlu_2_ NAM for clinical development would need to be far more potent and have improved brain homogenate binding to provide efficacy in AHL at a much lower dose. However, like M_4_ PAMs, an mGlu_2_ NAM mechanism could also address cognitive deficits, psychosis/behavioral symptoms and depression in not only schizophrenia and Alzheimer’s disease, but also Parkinson’s disease as this is not a cholinergic mechanism that might exacerbate motor symptoms.

**Figure 4:**
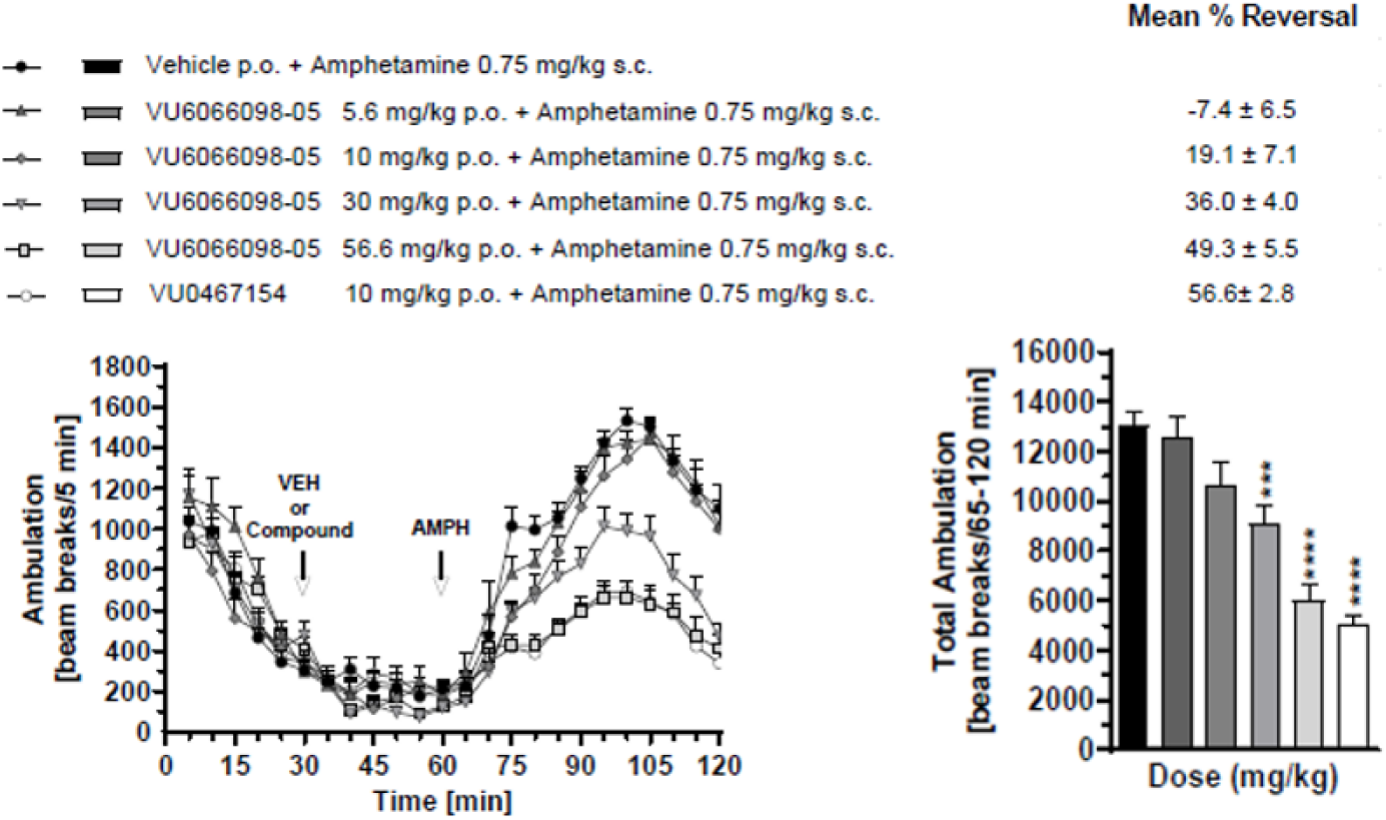
NAM **7** produced antipsychotic-like activity in the amphetamine (0.75 mg/kg, s.c.)-induced hyperlocomotion rat model. NAM **7** dose-dependently (5.6–56.6 mg/kg, p.o.) reversed amphetamine-induced hyperlocomotion (Left figure: time course of changes in ambulation [2-way ANOVA, treatment effect: F_5, 1464_ = 93.49, *p* < 0.001; treatment x time interaction: F_115, 1464_ = 6.085, *p* < 0.001]. Right figure: total ambulation [F_5, 61_ = 24.43, *p* < 0.001, *** *p* < 0.005 vs vehicle + amphetamine;****p < 0.0001 vs vehicle + amphetamine (1-way ANOVA followed by Dunnett’s test)]. *N* = 8–12 rats/group. VU0467154 (white symbols and bar) is a selective M_4_ PAM that served as a positive control in this assay.

### Cognition

All five of the mGlu_2_ NAMs **2**-**6** have demonstrated varying degrees of procognitive efficacy in both rat and nonhuman primate cognition models.^22–25,31^ Moreover, there is an aberrant expression of mGlu_2_ in vulnerable neurons in AD patients, indicating the target is present throughout the course of the disease, unlike the degenerated cholinergic system.^36^ We previously evaluated our mGlu_2_ NAMs in rat novel object recognition (NOR),^25^ a preclinical model of recognition memory enhancement, and the PK/PD relationship was once again linked to total brain concentration. With the improved mGlu_2_ NAM **7**, we bench-marked efficacy in rat NOR. Rats were pretreated with either a dose of the mGlu_2_ NAM **7** or vehicle prior to NOR training and then tested for recognition memory at 24 h (long-term memory, LTM) after training. Here (Figure 5), a robust improvement in recognition index was produced by NAM **7** at doses of 3 and 10 mg/kg p.o., with the MED at 3 mg/kg (total brain levels were 1.2 μM, or 10.5-fold the rat IC_50_, whereas free brain levels were 15.9 nM, or 0.14-fold the rat IC_50_). Thus, the PK/PD relationship with total brain seems to be retained.

**Figure 5:**
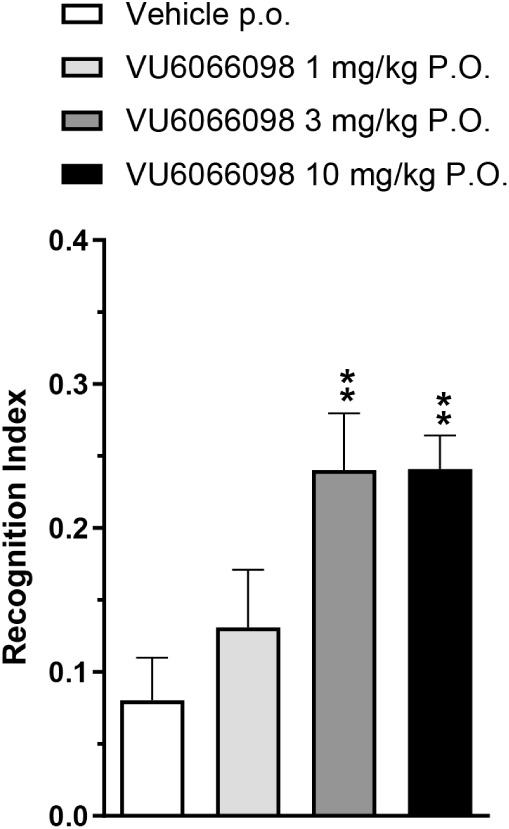
mGlu_2_ NAM **7** dose-dependently enhances the recognition memory in rats as assessed in a novel object recognition task. Pretreatment with 1, 3, and 10 mg/kg of NAM **7** (p.o. 10% Tween 80 in water) 1 h prior to exposure to identical objects significantly enhanced recognition memory assessed 24 h later (F_3, 65_ = 5.798, *p* < 0.01; **p < 0.01 Dunnett *post hoc* test). Minimum effective dose (MED) is 3 mg/kg. *N* = 17–18/group of male Sprague–Dawley rats.

In addition, the NAM **7** was evaluated for enhancement of the acquisition of contextual fear conditioning behavior (CFC) in rats (Figure 6). Contextual fear conditioning represents a classic associative learning and LTM task in which the response to an aversive foot shock stimulus becomes associated with a specific neutral context or testing environment; acquisition of this task is dependent on intact hippocampal functions.^35^ Using a one-shock paradigm, which resulted in ∼15% freezing, rats pretreated with 0.1, 0.3, and 1 mg/kg of NAM **7** (p.o. 10% Tween 80 in water) 1 h prior to the conditioning session, exhibited a dose-dependent enhancement in the acquisition of contextual fear conditioning behaviors as shown by increased percent freezing behaviors that were measured 24 h later under drug-free conditions in the same environmental context. NAM **7** dose-dependently enhanced the acquisition of CFC, with significant increases in percent freezing observed at the 0.3 and 1 mg/kg doses p.o. With the MED at 0.3 mg/kg (total brain levels were 46 nM, or 0.41-fold the rat IC_50_, whereas free brain levels were 0.62 nM, or 0.005-fold the rat IC_50_). At the 1 mg/kg dose, total brain levels were 318 nM, or 2.83-fold the rat IC_50_, whereas free brain levels were 4.29 nM, or 0.038-fold the rat IC_50_. The PK/PD relationship with total brain remains consistent across cognitive PD assays.

**Figure 6:**
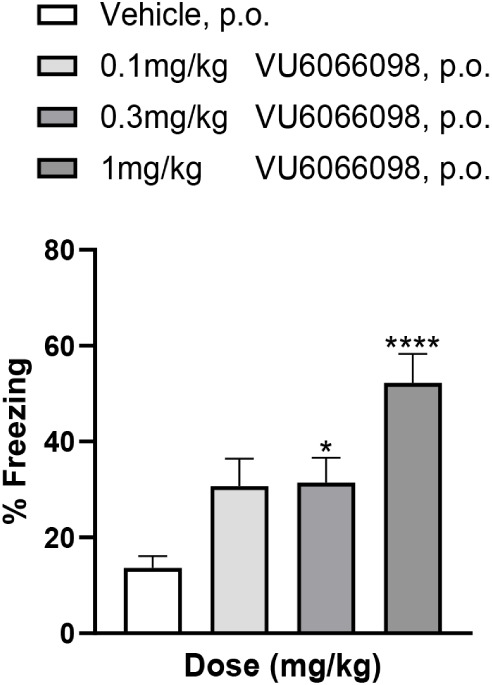
NAM **7** dose-dependently enhanced the acquisition of contextual fear conditioning (CFC) as denoted by increased percent freezing behavior in rats (F_3, 38_ = 10.59, *p* < 0.0001; *p < 0.05 vehicle vs 0.3 mg/kg and ****p < 0.0001 vehicle vs 1 mg/kg Dunnett *post hoc* test). Minimum effective dose (MED) is 0.3 mg/kg. *N* = 9–12/group of male Sprague–Dawley rats.

Many military veterans who experienced blast-related traumatic brain injuries (TBI) suffer from chronic cognitive and mental health problems including post-traumatic stress disorder (PTSD).^37,38^ Rats exposed to repetitive low level blast injury exhibit chronic PTSD-related behavioral traits.^39^ These traits can be reversed with the mGlu_2/3_ receptor antagonists BCI-838 and LY341495.^40^ In addition, mGlu_2_, but not mGlu_3_, expression is increased in brain at 43–52 weeks post blast, a time when the behavioral phenotype is established.^40^ This led us to examine whether the selective mGlu_2_ NAM **7** might also reverse these symptoms. Blast-exposed rats were treated at 8 months after exposure. IP doses of 10 mg/kg NAM **7** or vehicle (10% Tween 80) were given to blast-exposed rats on two consecutive days (days 1 and 2), while a group of sham-exposed rats were treated with vehicle alone. Two days later (day 4) rats were trained in a NOR task and then tested for recognition memory at 1 h (short-term memory, STM) or 24 h (LTM) after training. As shown in Figure 7A–B, NAM **7** reversed blast-induced deficits in the NOR task in both STM and LTM. To determine whether the response was sustained, we retested animals 30 days after the last drug administration. For this test, the novel object from the LTM testing at 24 h was replaced with a different novel object. As shown in Figure 7C, novel object recognition memory was still restored in blast-exposed rats treated with NAM **7** even 30 days after drug administration.

**Figure 7:**
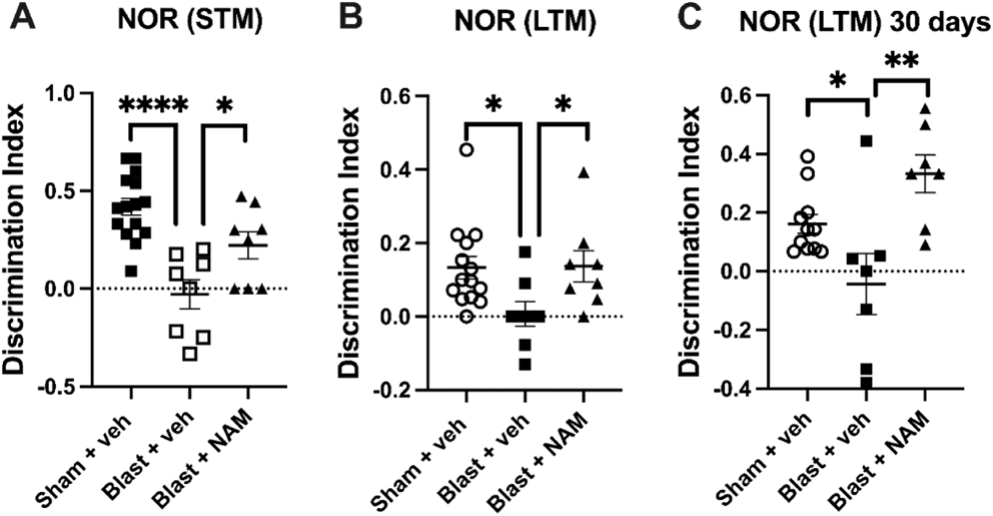
Control (sham-exposed) rats treated with vehicle (*N* = 15) and blast-exposed treated with vehicle (*N* = 8) or NAM **7** (*N* = 8), were trained in a NOR task and tested 1 h (A) or 24 h (B) later for recognition memory vs a novel object, and a discrimination index was calculated. One-way ANOVAs indicated significant between group effects (F_2, 22_ = 7.203, *p* = 0.0039 for STM; F_2, 28_ = 15.25, *p* < 0.0002 for LTM; * *p* < 0.05, **** *p* < 0.001, Fisher LSD). In panel (C), rats from panels (A) and (B) were tested 30 days after the last drug administration. A one-way ANOVA indicated significant between group effects (F_2, 22_ = 7.203, *p* = 0.0039; * *p* < 0.05, ** *p* < 0.01, Fisher LSD).

## Conclusions

A next-generation mGlu_2_ NAM *in vivo* tool compound, representing a novel, structurally distinct chemotype, demonstrated robust efficacy in rat preclinical models of antidepressant- and antipsychotic-like activity and enhancement of short and long-term recognition memory. PK/PD relationships suggests that total brain concentrations drove efficacy in rat forced swim and rat NOR assays, while free brain concentrations drove efficacy in AHL. In addition, the therapeutic potential of the mGlu_2_ NAM mechanism was extended into rat blast-related traumatic brain injuries (TBI), wherein the NOR efficacy persisted 30 days after drug administration. These data provide compelling evidence for the therapeutic potential of mGlu_2_ NAMs in Major depressive disorder, schizophrenia, Alzheimer’s disease, Parkinson’s disease and traumatic brain injury. Further optimization of NAM **7** toward a clinical development candidate is underway and will be reported in due course.

## Supplementary Material



## Notes

Additional experimental details; methods for the synthesis and characterization of all compounds; *in vitro* and *in vivo* DMPK protocols; supplemental figures (PDF)

## Abbreviations

K_p_: Plasma:brain partitioning coefficient
AD: Alzheimer’s disease
MED: Minimum effective dose
AHL: Amphetamine-induced hyperlocomotion
