# Supplementary material for: Discovery of VU6066098: A Selective and CNS-Penetrant mGlu2 NAM with Robust Antidepressant‑, Antipsychotic‑, and Procognitive-like Activity in Rodents
Source: ACS Chem Neurosci. 2026 Jun 23;17(14):2730–8. doi: 10.1021/acschemneuro.6c00324 (PMC13377601; doi:10.1021/acschemneuro.6c00324)
Supplement: Supplementary file 1 [file cn6c00324_si_001.pdf]

Discovery of VU6066098: A selective and CNS penetrant mGlu$_2$ NAM with robust antidepressant-, antipsychotic- and pro-cognitive-like activity in rodents

Jeremy S. Coleman,[1,2¥*] Rory A. Capstick,[1,2,5] Sichen Chang,[1,2,5] Derek J. Rios,[1,2,5] Michael Bubser, [1,2,5] Analisa D. Thompson Gray,[1,2,5] Irene Zagol-Ikapitte,[1,2,5] Srinivasan Krishnan,[1,2,5] Hyekyung P. Cho,[1,2,5] Alice L. Rodriguez[1,2,5], Colleen M. Niswender,[1,2,4,5, 11, 12] Olivier Boutaud,[1,2,5] Georgina Perez Garcia,[6,7] Gregory A. Elder,[7,8,9,10], Darren W. Engers,[1,2,5] Carrie K. Jones*[1,2,4,5] and Craig W. Lindsley*[1,2,3,4,5]

Affiliation:

[1]Warren Center for Neuroscience Drug Discovery, Vanderbilt University, Nashville, TN 37232, USA
[2]Department of Pharmacology, Vanderbilt University School of Medicine, Nashville, TN 37232, USA
[3]Department of Chemistry, Vanderbilt University, Nashville TN 37232, USA
[4]Vanderbilt Institute of Chemical Biology, Vanderbilt University, Nashville, TN 37232, USA
[5]Vanderbilt Institute for Therapeutic Advances, Vanderbilt University, Nashville, TN 37232, USA
[6] Research and Development Service, James J. Peters Department of Veterans Affairs Medical Center, 130 West Kingsbridge Road, Bronx, New York 10468, USA
[7] Department of Neurology, Icahn School of Medicine at Mount Sinai, One Gustave Levy Place, New York, New York 10029, USA
[8] Department of Psychiatry, Icahn School of Medicine at Mount Sinai, One Gustave Levy Place, New York, New York 10029, USA
[9] Mount Sinai Alzheimer's Disease Research Center and Ronald M. Loeb Center for Alzheimer's Disease, Icahn School of Medicine at Mount Sinai, One Gustave Levy Place, New York, New York 10029, USA
[10] Neurology Service, James J. Peters Department of Veterans Affairs Medical Center, 130 West Kingsbridge Road, Bronx, New York 10468, USA
[11] Vanderbilt Brain Institute, Vanderbilt University, Nashville, TN 37240,USA
[12] Vanderbilt Kennedy Center, Vanderbilt University, Nashville, TN 37203, USA

Please add Vanderbilt Brain Institute, Vanderbilt University, and Vanderbilt Kennedy Center, Vanderbilt University Medical Center to my affiliations—thanks!

*To whom correspondence should be addressed at carrie.jones@vanderbilt.edu and craig.lindsley@vanderbilt.edu

TABLE OF CONTENTS

Procedures for Biological Experiments S2
General Methods S14
Synthetic Procedures and Spectra S15
Eurofins SafetyScreen44 .... S18

Supplemental Figures..S19

Molecular Pharmacolgy Methods

Assessment of Cellular Potency of $mGlu_2$ compounds in Thallium Flux Assay

Human embryonic kidney 293 (HEK293) cell lines stably co-expressing human or rat $mGlu_2$ and G protein-coupled inwardly rectifying potassium (GIRK) channels were maintained in growth medium containing DMEM/F12 containing 10% FBS, 20 mM HEPES, 1 mM sodium pyruvate, 2 mM L-glutamine, non-essential amino acids mixture, 1X Antibiotic/Antimycotic, 700 µg/mL G418, and 0.6 µg/mL puromycin. The day before the assay, $mGlu_2$ /GIRK cells were plated into 384 well, black-walled, clear-bottom poly-D-lysine coated plates at a density of 15,000 cells/20 µL/well in DMEM containing 10% dialyzed FBS, 20 mM HEPES, 1 mM sodium pyruvate, and 1X Antibiotic/Antimycotic. The cells were incubated overnight in 37 °C humidified incubators in the presence of 5% CO_2.
The next day, GIRK assay buffer (Hank's balanced salt solution (HBSS), 20 mM HEPES, and 4.16 mM sodium bicarbonate, pH 7.4) was prepared and used to dilute compounds and Thallos-acetomethoxyester (Thallos-AM, Ion Biosciences), a fluorescent thallium indicator. Compounds were serially diluted 1:3 into 10-point concentration response curves in DMSO using a Bravo Liquid Handler (Agilent, Santa Clara, CA), transferred to a 384 well daughter plates using an Echo acoustic liquid handler (Beckman Coulter, Indianapolis, Indiana), and diluted in assay buffer to a 2X final concentration. A 2X dye solution (1.36 µM) was prepared by mixing a 2.97 mM Thallos-AM stock in DMSO with 10% (w/v) pluronic acid F-127 in a 1:1 ratio in assay buffer. The agonist plates were prepared using glutamate concentrations for the EC_{80} and EC_{Max} responses to a 5X final concentration in thallium stimulation buffer (125 mM sodium bicarbonate, 1.8 mM $CaSO_4$, 1 mM $MgSO_4$, 5 mM glucose, 12.5 mM Tl_2SO_4, 10 mM HEPES, pH 7.4). Using a microplate washer (BioTek, Winooski, VT), cells were washed with GIRK assay buffer 4 times to remove media. After the final wash, 20 µL of assay buffer remained in the cell plates. Immediately, 20 µL of the 2X dye solution (final 0.68 µM) was added to each well of the cell plate using a Multidrop Combi dispenser (Thermo Fisher, Waltham, MA). After cells were incubated with the dye solutions for 60 min at room temperature, the dye solutions were removed and replaced with GIRK assay buffer using a microplate washer, leaving 20 µL of assay buffer in the cell plate. The compound, agonist, and cell plates were placed inside the Functional Drug Screening System (FDSS 7000 or µCell kinetic imaging plate reader, Hamamatsu, Japan) to measure the thallium flux using a double add protocol. After establishment of a fluorescence baseline (excitation, 480 nm; emission, 530 nm), 20 µL (2x) of test compound was added to the cells at 2 seconds and the response was measured. 140 seconds later, 10 µL (5x) of an EC_{80} concentration of glutamate or vehicle in thallium stimulus buffer was added to the cells, and the response of the cells was measured for an additional 158 seconds. Multiple reference wells containing no compound (DMSO vehicle) received either no glutamate (for a baseline reference), EC_{80} glutamate, or EC_{Max} glutamate (for normalization to maximum response). Fluorescence was measured throughout the experiment at a frequency of 1 measurement per 2 seconds before the glutamate/thallium addition and a frequency of 1 measurement per 1 second after the glutamate/thallium addition. Each compound concentration series occurred one time in each plate, and replicates from two or three plates were used in each experimental run. Thallium solutions and plastic solid waste were handled and disposed of according to guidelines from the Vanderbilt University Chemical Safety department. Data were normalized using a static ratio function (F/F_0) by dividing every fluorescent measurement by the initial fluorescent value for the corresponding well. The increase in signal resulting from the glutamate/thallium addition of the second add was measured by determining the slope from the time window of 145 seconds to 155 seconds. The average of all baseline slopes (no compound, no glutamate) was determined and this value was subtracted from all other slope values. The average of all EC_{Max} slopes was determined, and this value was used to normalize the baseline-corrected slopes to a percent max value ($\%E_{Max}$).

Compound-evoked decreases in thallium flux response in the presence of glutamate EC_{80} agonist were determined as negative allosteric modulator (NAM) activity, and potency (IC_{50}) and maximum responses (% Glu_{Max}) of compounds were determined using a four-parameter logistical equation using GraphPad Prism (La Jolla, CA) or the Dotmatics software platform (Woburn, MA):

$$y = bottom + \frac{top - bottom}{1 + 10^{(LogEC50 - A)Hillslope}}$$

where A is the molar concentration of the compound; bottom and top denote the lower and upper plateaus of the concentration-response curve; HillSlope is the Hill coefficient that describes the steepness of the curve; and EC_{50} is the molar concentration of compound required to generate a response halfway between the top and bottom.

Assessment of Cellular Potency of $mGlu_2$ compounds in cAMP Assay

Human $mGlu_2$ cAMP antagonist mode assay was performed at LeadHunter Services of Eurofins DiscoverX Corporation (Fremont, CA)

mGlu Receptor Subtype Selectivity Screening Assays

The mGlu receptor subtype selectivity of compounds was determined using thallium flux or calcium mobilization assays using stable cell lines expressing the respective human or rat mGlu receptor subtypes. Thallium flux assay was performed for rat $mGlu_4$ and human/rat $mGlu_3$ as exactly described above. Calcium mobilization assays were performed for human $mGlu_4$ and human/rat $mGlu_{1,5,7,8}$.
Briefly, the day before calcium assay, respective mGlu subtype expressing cells were plated in black-walled, clear-bottomed, poly-D-lysine coated 384-well plates at a density of 20,000 cells/well in 20 µL of assay medium (DMEM supplemented with 10% dialyzed FBS, 20 mM HEPES, and 1 mM sodium pyruvate); Human and rat $mGlu_5$-HEK293A stable cells, human and rat $mGlu_7$ co-expressing with the promiscuous G protein $G_{\alpha15}$-HEK293 Cells . For tetracycline-inducible human and rat $mGlu_1$ TREx293 cells, cells were plated in the assay medium containing 10ng/mL tetracycline to induce $mGlu_1$ expression. Human and Rat $mGlu_8$/$G_{\alpha15}$-HEK293 cells were plated at a density of 15,000 cells/well in 20 µL and human $mGlu_4$ co-expressing chimeric G protein G_{qi5}-CHO cells were plated at 30,000 cells. The cells were incubated overnight at 37 ºC in the presence of 5% CO_2. The next day, calcium assay buffer (Hank's balanced salt solution (HBSS), 20 mM HEPES, 2.5 mM Probenecid, 4.16 mM sodium bicarbonate was prepared to dilute compounds, agonists, and Fluo-4-acetomethoxyester (Fluo-4-AM, Ion Biosciences), fluorescent calcium indicator dye. Compounds were serially diluted 1:3 into 10-point concentration response curves in DMSO using a Bravo Liquid Handler (Agilent, Santa Clara, CA), transferred to a 384 well daughter plates using an Echo acoustic liquid handler (Beckman Coulter, Indianapolis, Indiana), and diluted in assay buffer to a 2X final concentration. The agonist plates were prepared using glutamate (for $mGlu_{1,4,5,8}$) and DL-AP4 (for $mGlu_7$) concentrations for the EC_{20}, EC_{80} and EC_{Max} responses by diluting in assay buffer to a 5X final concentration. A 2X dye solution (2.3 µM) was prepared by mixing a 2.3 mM Fluo-4-AM stock in DMSO with 10% (w/v) pluronic acid F-127 in a 1:1 ratio in assay buffer. Using a microplate washer (BioTek, Winooski, VT), cells were washed with assay buffer 4 times to remove media. After the final wash, 20 µL of assay buffer remained in the cell plates. Immediately, 20 µL of the 2X dye solution (final 1.15 µM) was added to each well of the cell plate using a Multidrop Combi dispenser (Thermo Fisher, Waltham, MA). After cells were incubated with the dye solutions for 45 min at 37 °C in the presence of 5% CO_2, the dye solutions were removed and replaced with assay buffer using a microplate washer, leaving 20 µL of assay buffer in the cell plate. The compound, agonist, and cell plates were placed inside the Functional Drug Screening System (FDSS 7000 or µCell kinetic imaging plate reader, Hamamatsu, Japan) to measure the calcium flux. Assays were run at 37 °C. A triple add protocol was used to measure Ca kinetics. Briefly, after establishment of a fluorescence baseline for 2 seconds (excitation, 480 nm; emission, 530 nm), 20 µL of test compound was added to the cells and the response was measured for 142 seconds. This was followed by the addition of 10 µL (5X) of an EC_{20} concentration of

glutamate/DL-AP4 agonist, and the response of the cells was measured for 125 seconds. A third addition occurred by adding 12 uL (5X) of an EC_{80} concentration of glutamate/DL-AP4 agonist and the response of the cells was measured for 90 seconds. Vehicle (0.6 % DMSO) in assay buffer was added to the control wells at the 1st add for measuring glutamate EC_{20}, EC_{80}, and EC_{Max} responses. Calcium fluorescence was recorded as fold over basal fluorescence and raw data were normalized to the maximal response to glutamate/DL-AP4 agonist (EC_{Max}). Compound-evoked increases in calcium response in the presence of EC_{20} agonist were determined as positive allosteric modulator (PAM), and potency (EC_{50}) and maximum potentiation responses (% E_{Max}) of compounds were determined using a four-parameter logistical equation using GraphPad Prism (La Jolla, CA) or the Dotmatics software platform (Woburn, MA). Compound-evoked decreases in calcium response in the presence of EC_{80} agonist were determined as NAM, and potency (IC_{50}) and maximum inhibitory response were also determined. Compounds with no measurable activity are designated as inactive (>10 μM)

Drug Metabolism Methods:

In vitro

Plasma protein binding and Brain homogenate binding: Determination of fraction unbound (f_u) in plasma was conducted in vitro via equilibrium dialysis using HTDialysis (HTD) membrane plates. The top half of the plate was filled with 100 uL of Dubelco's Phosphate Buffered Saline, pH 7.4 (DPBS). Compounds were diluted into plasma from each species (5 μM final concentration), which was aliquoted in triplicate to the 'bottom half' of the prepared HTD plate wells. The HTD plate was sealed and incubated for 6 hours at 37 °C. Following incubation, each well (both top and bottom halves) was transferred (20 μL) to the corresponding wells of a 96-shallow-well (V-bottom) plate. The daughter plates were then matrix-matched (DPBS side wells received equal volume of plasma, and plasma side wells received equal volume of DPBS), and extraction solution (120 μL; acetonitrile containing 50 nM carbamazepine as IS) was added to all wells of both daughter plates to precipitate protein and extract the test article. The plates were then sealed and centrifuged (3500 rcf) for 10 minutes at ambient temperature. Supernatant (60 μL) from each well of the daughter plates was then transferred to the corresponding wells of new daughter plates (96-shallow-well, V bottom) containing water (Milli-Q, 60 μL/well), and the plates were sealed in preparation for LC-MS/MS analysis (see LC-MS/MS analysis method below).

The unbound fraction (f_u) was calculated following the equation below, and mean values for each species were calculated from 3 replicates.

A similar approach was used to determine the degree of brain homogenate binding, which employed the same methodology and procedure with the following modifications: 1) a final compound concentration of 1 μM was used, 2) naïve rat brains were homogenized in DPBS (1:3

composition of brain: DPBS, w/w) using a Mini-Bead Beater™ machine in order to obtain brain homogenate, which was then treated in the same manner as the plasma samples in the previously described plasma protein binding assay. Fraction unbound for both plasma and brain samples was determined using Equation 4.

$$f_u = \frac{Conc_{buffer}}{Conc_{plasma}}$$

Equation 4 Determination of fraction unbound in plasma.

The diluted fraction unbound (f_u2) in brain was calculated in the same manner by using brain homogenate rather than plasma. Undiluted fraction unbound for the brain was calculated using Equation 5

$$f_u = \frac{1/4}{\left\{\left(\frac{1}{fu2}\right) - 1\right\} + 1/4}$$

Equation 5 Determination of fraction unbound in brain. F_u2 represents the diluted fraction unbound.

Intrinsic clearance: Human or rat hepatic microsomes (0.5 mg/mL) and 1 µM test compound were incubated in 100 mM potassium phosphate pH 7.4 buffer with 3 mM $MgCl_2$ at 37 °C with constant shaking. After a 5 min preincubation, the reaction was initiated by the addition of NADPH (1 mM). At selected time intervals (0, 3, 7, 15, 25, and 45 min), aliquots were taken and subsequently placed into a 96-well plate containing cold acetonitrile with internal standard (50 ng/mL carbamazepine). Plates were then centrifuged at 3000 rcf (4 °C) for 10 min, and the supernatant was transferred to a separate 96-well plate and diluted 1:1 with water for LC/MS/MS analysis. The *in vitro* half-life ($_{1/2}$, min, Eq. 1), intrinsic clearance (CL_{int}, mL/min/kg, Eq. 2), and subsequent predicted hepatic clearance (CL_{hep}, mL/min/kg, Eq. 3) was determined employing the following equations:

(1) $T_{1/2} = \frac{Ln(2)}{K}$

where k represents the slope from linear regression analysis of the natural log percent remaining of a test compound as a function of incubation time

$$(2)\ CL_{int} = \frac{0.693}{in\ vitro T_{1/2}}\ x\ \frac{mL\ incubation}{mg\ microsomes}\ x\ \frac{45\ mg\ microsomes}{gram\ liver}\ x\ \frac{20^{a}\ gram\ liver}{kg\ body\ wt}$$

[a]scale-up factors: of 20 (human) or 45 (rat)

$$(3)\ CL_{hep} = \frac{Q_h \cdot CL_{int}}{Q_h + CL_{int}}$$

where Q_h (hepatic blood flow, mL/min/kg) is 21 (human) or 70 (rat).

LC/MS/MS Bioanalysis of Samples from Plasma Protein Binding and Intrinsic Clearance Assays:

Samples were analyzed on a Thermo Electron TSQ Quantum Ultra triple quad mass spectrometer (San Jose, CA) via electrospray ionization (ESI) with two Themo Electron Accella pumps (San Jose, CA), and a Leap Technologies CTC PAL autosampler (Carrboro, NC). Analytes were separated by gradient elution on a dual column system with two Thermo Hypersil Gold (2.1 x 30 mm, 1.9 µm) columns (San Jose, CA) thermostated at 40 °C. HPLC mobile phase A was 0.1% formic acid in water and mobile phase B was 0.1% formic acid in acetonitrile. The gradient started at 10% B after a 0.2 min hold and was linearly increased to 95% B over 0.8 min; hold at 95% B for 0.2 min; returned to 10% B in 0.1 min. The total run time was 1.3 min and the HPLC flow rate was 0.8 mL/min. While pump 1 ran the gradient method, pump 2 equilibrated the alternate column isocratically at 10% B. Compound optimization, data collection, and processing were performed using Thermo Electron's QuickQuan software (v2.3) and Xcalibur (v2.0.7 SP1).

***In vivo* DMPK experimental:**

Determination of brain to plasma ratio:

Animal care and use

All animal study procedures were approved by the Institutional Animal Care and Use Committee and were conducted in accordance with the National Institutes of Health regulations of animal care covered in Principles of Laboratory Animal Care (National Institutes of Health).

In-life phase

For determination of the brain over plasma ratio (K_p), compounds were formulated in 8% ethanol, 32% PEG400 and 60% DMSO (*v/v/v*) and administered as a single 0.2 mg/kg IV dose (1 mL/kg) to male, Sprague Dawley rats (n = 1) via injection into a surgically-implanted jugular vein catheter. At 15 min post dosing, blood sample was collected into chilled, K_2EDTA anticoagulant-fortified tube and immediately placed on wet ice. The blood sample was then centrifuged (1700 rcf, 5 minutes, 4 ˚C) to obtain plasma sample. At the same post-administration time point, whole brain sample was obtained by rapid dissection, rinsed with PBS, and immediately frozen in individual tissue collection box (dry ice). All brain and plasma samples were stored at -80 ˚C until analysis by LC-MS/MS.

Sample Analysis: Concentrations in plasma and brain homogenates were quantified by liquid chromatography tandem mass spectrometry (LC-MS/MS). Whole brains were homogenized in 3 mL of 70:30 IPA:water in a mini bead beater for 3 min, and centrifuged at 3,500 g for 5 min. 5 uL of the supernatant was diluted in 15 uL of blank plasma for quantification of the analytes. Plasma samples were centrifuged at 3,500 g for 5 min. A standard curve was generated by diluting the analytes DMSO stocks with blank plasma to obtain a final concentration of 10,000 ng/ml followed by a serial dilution down to 0.5 ng/ml. Quality controls were generated by a serial dilution of the 5,000 ng/ml standard curve solution in blank plasma to obtain 3 concentrations of 500, 50, and 5 ng/ml. 20 uL of brain diluted in plasma, plasma, blank plasma, standard curve and QC samples were loaded in a V-bottom 96-well plate. 120 uL of acetonitrile containing 0.05 uM carbamazepine (internal standard) was added to each well and the plate was centrifuged at 3,500 g for 5 min. 60 uL of the supernatant of each well (protein free) was transferred to a new 96-well plate containing 60 uL of water. The plates were sealed for analysis by LC-MS/MS.

Plasma and brain tissue samples originating from *in vivo* studies were analyzed by electrospray ionization using an AB Sciex Q-TRAP 5500 (Foster City, CA) that was coupled to a Shimadzu LC-20AD pump (Columbia, MD) and a Leap Technologies CTC PAL auto-sampler (Carrboro, NC). Analytes were separated by gradient elution using a C18 column (3 x 50 mm, 3 mm; Fortis Technologies Ltd, Cheshire, UK) that was thermostated at 40 °C. HPLC mobile phase A was 0.1%

formic acid in water (pH unadjusted); mobile phase B was 0.1% formic acid in acetonitrile (pH unadjusted). A 10% B gradient was held for 0.2 min and was linearly increased to 90% B over 0.8 min, with an isocratic hold for 0.5 min, before transitioning to 10% B over 0.05 min. The column was re-equilibrated (1 min) before the next sample injection. The total run time was 2.55 min, and the HPLC flow rate was 0.5 ml/min. The source temperature was set at 500 °C, and mass spectral analyses were performed using a Turbo-Ion spray source in positive ionization mode (5.0-kV spray voltage) and using multiple-reaction monitoring of transitions specific for the analytes. All data were analyzed using AB Sciex Analyst 1.5.1 software.

Brain plasma concentration ratio (K_p) was calculated by dividing brain concentration by plasma concentration for each animal. Unbound brain to unbound plasma concentration ratio ($K_{p,uu}$) is calculated using the following formula: $K_{p,uu}$ = (Brain ng/g x brain fu)/(plasma ng/ml x plasma fu).

Pharmacokinetic profiles in rats following oral single escalating doses

Single escalating oral dosing in Sprague-Dawley rats was performed at Frontage Laboratories according to their non-GLP Standard Operating Procedure and IACUC protocols. In short, compounds were formulated in 10% Tween 80 in water and dosed at 10 mg/kg. At different times, arterial blood was collected from a femoral artery catheter, and compound concentration was determined in plasma by LC-MS/MS following their non-GLP protocol. PK parameters were determined using Phoenix WinNonlin software (version 6.3).

***In vitro* determination of blood-brain barrier penetration potential**

Blood-brain barrier penetration was determined using MDR1-MDCK cell monolayers by Absorption Systems, following their protocol. In short, compounds were incubated at 5 mM final concentration on one side of the cell monolayer for 2 hours. Compounds concentration on either side of the monolayer was determined by LC-MS/MS and apparent permeability and efflux ratio were determined as described in Wang, Q. et al.[1]

Behavioral Studies Methods

Animals. Adult male Sprague-Dawley rats weighing 275 - 299 g were obtained from Envigo (Indianapolis, Indiana) for the behavioral and pharmacokinetic studies. Rats were group-housed (2 - 3 animals per cage) under a 12 h light/12-h dark cycle with food and water *ad libitum*. For the

amphetamine hyperlocomotion and the forced swim assays, rats were fasted overnight prior to the testing day. For the novel object recognition and contextual fear conditioning assays, rats were not food-deprived to avoid impact of food deprivation on cognitive performance.

All animal studies were approved by the Vanderbilt University Institutional Animal Care and Use Committee and followed the guidelines set forth by the *Guide for the Care and Use of Laboratory Animals*.

Novel Object Recognition Test. This study was carried out during the inactive phase of the rats (light phase) and all training and testing procedures were performed under red light conditions. The Novel Object Recognition (NOR) task was performed as described previously. For 2 consecutive days, rats were individually habituated for 10 min in an empty black composite chamber (40 cm × 64 cm × 33 cm). On the following day, vehicle (10% Tween 80 in milliQ water) or VU6066098 (1, 3, or 10 mg/kg) were administered to the animals by oral gavage (p.o.), then rats were returned to their home cage. 60 min later, rats were placed for 10 min in the novel object recognition chamber containing two identical objects (either two 590-mL Gatorade® bottles filled with water or two weighted stainless steel pipette sterilization boxes [5.1 cm × 5.1 cm × 23.5 cm] and subsequently returned to their home cage. 24 h later, rats were returned to the chamber in a configuration where one of the familiar (previously exposed) objects remained while the other familiar object was replaced by a novel object. For each treatment group, the object type serving as familiar and novel was counterbalanced in a pseudo-randomized fashion. The behavior of each rat exploring the familiar and novel objects was video recorded for 3 min for subsequent analysis. Object exploration was defined as sniffing of the objects that was accompanied by active vibrissae movements. The time spent exploring each object was scored by an observer blinded to both the experimental treatment condition and the object types representing novel and familiar objects.

Data Analysis. For each rat the recognition index was calculated as follows:

$$Recognition\ Index\ = \frac{(\text{time exploring Novel Object} - \text{time exploring Familiar Object})}{(\text{time exploring Novel Object} + \text{time exploring Familiar Object})}$$

Thus, recognition indices of 1.0, 0, and -1.0 indicate exclusive exploration of Novel Object, equal exploration of Novel and Familiar Object, and exclusive exploration of Familiar Object,

respectively. Data were analyzed by one-way ANOVA followed by Dunnett's test using GraphPad Prism 9 and $p \leq 0.05$ was considered to represent statistical significance.

Reversal of Amphetamine-Induced Hyperlocomotion. Adult male Harlan Sprague Dawley rats were tested in SmartFrame Open Field locomotor activity test chambers (Kinder Scientific, Poway, CA) to automatically record locomotor activity. All rats were habituated in locomotor activity enclosures for 30 min, followed by pretreatment by oral gavage for an additional 30 min with either vehicle, a dose of NAM **7** (5.6, 10, 30 , 56.6 mg/kg) or the positive comparator M_4 PAM VU0467154 (10 mg/kg). Thirty minutes later, rats were injected subcutaneously with a dose of 0.75 mg/kg amphetamine and then monitored for an additional 60 min. Changes in locomotor activity were recorded for a total of 120 min. Locomotor data were expressed as the number of photobeam breaks/5 min intervals across the 120-min test session or as the total ambulation, calculated as sum of photobeam beam breaks from the time of amphetamine administration (60 min) until the end of the study (120 min). Time course data were analyzed by two-way ANOVA with main effects of treatment and time; changes in total ambulation were analyzed by one-way ANOVA followed by Dunnett's post hoc test (GraphPad Prism 7 [GraphPad Software, San Diego, CA]). For all tests, $p \leq 0.05$ was considered to represent statistical significance. Finally, percent reversal data were calculated in Microsoft Excel using the following formula: Percent Reversal = 100 - {[(total ambulation in individual animal from t = 60 to t = 120) / (mean total ambulation from t=60 to t=120 in the VAMP group)] * 100}. Mean percent reversal ± S.E.M. was calculated for each dose group using GraphPad Prism 7

Rat Forced Swim. Adult male Harlan Sprague Dawley rats were tested in Porsolt Forced Swim tanks (Stoeltling Co , Wood Dale, IL). All rats were habituated for 10 min each in the forced swim tanks containing 23-25°C water approximately 30 cm deep for two days prior to testing. On day three the rats were pretreated via oral gavage for 30 minutes with vehicle (10% Tween 80 in milliQ water), a dose of VU6066098 (1, 3 or 10 mg/kg) or subcutaneously with the positive comparator 10 mg/kg ketamine. Each rat was individually placed into separate swim tanks and allowed to swim for 6 minutes. All rat swim behaviors were video taped using a Sony video camera and manually scored by a staff member blinded to treatment. The duration of immobility

behavior was scored in seconds over the 6 min recorded testing time. Immobility was characterized a floating posture, in which each rat made only the minimal movements necessary to keep its head above water. Changes in immobility were analyzed by one-way ANOVA followed by Dunnett's post-hoc analysis (GraphPad Prism 10 [GraphPad Software, San Diego, CA]). For all tests, $p \leq 0.05$ was considered to represent statistical significance.

Contextual Fear Conditioning. Adult Male Sprague−Dawley rats were conditioned in sound-attenuating cubicle (Med Associates, St. Albans, VT). Rats were pretreated 1 hour prior to conditioning with either vehicle (p.o.) or a dose of NAM 7 (0.1, 0.3, 1 and 3 mg/kg, PO), then placed in the conditioning chambers with an 10% Vanilla as an olfactory cue and allowed to habituate for 210 seconds. After habituation, rats were exposed to 1 footshock (1-s, 0.5 mA) followed by 129 seconds of no stimuli and then were immediately returned to their home cage. Two rats were dosed and train simultaneously in two different chambers within the same room. Approximately 24 h after conditioning, rats were placed in the same chambers used on the conditioning day and exposed to 10% vanilla olfactory stimulus. During the testing day procedure rats were not administered compound treatment or shock stimulus. Contextual fear conditioning was assessed for a total of 240 seconds. Freezing behavior was defined as cumulative % percent freezing behavior over the testing period. Duration of freezing behavior was automated by VideoFreeze™ Video Fear Conditioning Software (Med-Associates Inc., St. Albans, VT) and manually recorded for analysis. All data are presented as means ± S.E.M. Data were analyzed by one-way ANOVA followed by Dunnett's test using GraphPad Prism 10 (GraphPad Software, San Diego, CA). For all tests, $p \leq 0.05$ was considered to represent statistical significance.

Rat Blast TBI

Animals

A total of 31 adult male Long Evans hooded rats (250–350 g; 10 weeks of age; Charles River Laboratories International, Wilmington, MA, USA) were used. All studies involving animals were approved by the Institutional Animal Care and Use Committee of the James J. Peters VA Medical Center. Studies were conducted in compliance with the Public Health Service policy on the

humane care and use of laboratory animals, the NIH Guide for the Care and Use of Laboratory Animals, and all applicable Federal regulations governing the protection of animals in research.

Blast overpressure exposure

Rats were exposed to overpressure injury using a shock tube at the James J. Peters VA Medical Center designed and constructed by Baker Engineering and Risk Consultants (San Antonio, TX). The instrument is approximately 6.4 m in length. The tube consists of a variable volume driver that permits control of the duration of the primary positive peak pressure wave independent of the overpressure peak. The end of the shock tube is fitted with an attenuator chamber that reduces ambient blast noise and suppresses reflected shock waves. Pressurized air was used for all experiments. A dual diaphragm spooler that holds two polyethylene terephthalate Mylar TM sheets (Du Pont, Wilmington, DE, USA) was used to control pressure differences between the driver and expansion section of the tube. To induce ‘detonation’, the pressure between the two diaphragms of the spooler was released via remotely controlled electronic valves causing both diaphragms to rupture simultaneously. The peak pressure at the end of the expansion chamber was determined with piezoresistive gauges specifically designed for pressure-time (impulse) measurements (Model 102M152, PCB, Piezotronics, Depew, NY, USA). All sensor data was collected and processed using Lab View2011 software (Austin, TX).

Individual rats were anesthetized using an isoflurane gas anesthesia system consisting of a vaporizer, gas lines and valves and an activated charcoal-scavenging system adapted for use with rodents. Rats were placed into a polycarbonate induction chamber, which was closed and immediately flushed with 5% isoflurane mixture in air for two minutes. Rats were placed into a cone-shaped plastic restraint device and mounted on a square grid. Head and body movement was restricted by harnesses that affixed the animal to the grid and restricted movement during the blast overpressure exposure without restricting breathing. Rats were randomly assigned to sham or blast conditions and were placed in the shock tube lying prone with the plane representing a line from the tail to the nose of the body in line with the longitudinal axis of the shock tube and the head placed more upstream. The total length of time under anesthesia including placement in the shock tube and execution of the blast procedure was typically less than 3 minutes. Blast-exposed animals

received 74.5 kPa peak overpressure exposures (equivalent to 10.8 psi, duration 4.8 ms, impulse 175.8 kPa*ms).

Animal housing

Animals were housed at a constant 21-22 ^{o}C temperature with rooms on a 12:12 hour light cycle with lights on at 7 AM. All subjects were individually housed in standard clear plastic cages equipped with Bed-O'Cobs laboratory animal bedding (The Andersons, Maumee, OH, USA) and EnviroDri nesting paper (Sheppard Specialty Papers, Milford, NJ, USA). Access to food and water was ad libitum. Subjects were housed on racks in random order to prevent rack position effects. Cages were coded to allow maintenance of blinding to groups during behavioral testing.

Behavioral testing

Novel object recognition (NOR). Rats were habituated to the arena (90 cm length x 60 cm width x 40 cm height) for 20 min, 24 h before training. On the training day, two identical objects were placed on opposite ends of the empty arena, and the rat was allowed to explore the objects freely for 7 min. After a 1-h delay, during which the rat was held in its home cage, one of the two familiar objects was replaced with a novel one, and the rat was allowed to freely explore the familiar and novel object for 5 min to assess short-term memory (STM). After a 24-h delay, during which the rat was held in its home cage, one of the two familiar objects was replaced with a novel one different from the ones used during the STM. The rat was allowed to freely explore the familiar and novel object for 5 min to assess long-term memory (LTM). Raw exploration times for each object were expressed in seconds. Object exploration was defined as sniffing or touching the object with the vibrissae or when the animal's head was oriented toward the object with the nose placed at a distance of less than 2 cm from the object. All sessions were recorded by video camera (Sentech, Carrollton TX, USA) and analyzed with ANYMAZE software (San Diego Instruments). In addition, offline analysis by an investigator blind to the blast-exposed status of the animals was performed. Objects to be discriminated were of different size, shape and color and were made of plastic or metal material. The objects consisted of a 330 ml soda can, a metal box, a cup and a

plastic tube. All objects were cleaned with 70% ethanol between trials. A discrimination index was calculated for the recognition memory testing with the formula: (time exploring the NO minus time exploring the FO)/(total exploration time) x 100.-

Experimental Procedures and Spectroscopic Data General.

All NMR spectra were recorded on a 400 MHz AMX Bruker NMR spectrometer. 1H and 13C chemical shifts are reported in δ values in ppm downfield with the deuterated solvent as the internal standard. Data are reported as follows: chemical shift, multiplicity (s = singlet, d = doublet, t = triplet, q = quartet, b = broad, m = multiplet), integration, coupling constant (Hz). Low resolution mass spectra were obtained on an Agilent 6120 or 6150 with ESI source. Method A: MS parameters were as follows: fragmentor: 70, capillary voltage: 3000 V, nebulizer pressure: 30 psig, drying gas flow: 13 L/min, drying gas temperature: 350 ºC. Samples were introduced via an Agilent 1290 UHPLC comprised of a G4220A binary pump, G4226A ALS, G1316C TCC, and G4212A DAD with ULD flow cell. UV absorption was generally observed at 215 nm and 254 nm with a 4 nm bandwidth. Column: Waters Acquity BEH C18, 1.0 x 50 mm, 1.7 um. Gradient conditions: 5% to 95% CH3CN in H2O (0.1% TFA) over 1.4 min, hold at 95% CH3CN for 0.1

min, 0.5 mL/min, 55 ºC. Method B: MS parameters were as follows: fragmentor: 100, capillary voltage: 3000 V, nebulizer pressure: 40 psig, drying gas flow: 11 L/min, drying gas temperature: 350 ºC. Samples were introduced via an Agilent 1200 HPLC comprised of a degasser, G1312A binary pump, G1367B HP-ALS, G1316A TCC, G1315D DAD, and a Varian 380 ELSD (if applicable). UV absorption was generally observed at 215 nm and 254 nm with a 4 nm bandwidth. Column: Thermo Accucore C18, 2.1 x 30 mm, 2.6 um. Gradient conditions: 7% to 95% CH3CN in H2O (0.1% TFA) over 1.6 min, hold at 95% CH3CN for 0.35 min, 1.5 mL/min, 45 ºC. High resolution mass spectra were obtained on an Agilent 6540 UHD Q-TOF with ESI source. MS parameters were as follows: fragmentor: 150, capillary voltage: 3500 V, nebulizer pressure: 60 psig, drying gas flow: 13 L/min, drying gas temperature: 275 ºC. Samples were introduced via an Agilent 1200 UHPLC comprised of a G4220A binary pump, G4226A ALS, G1316C TCC, and G4212A DAD with ULD flow cell. UV absorption was observed at 215 nm and 254 nm with a 4 nm bandwidth. Column: Agilent Zorbax Extend C18, 1.8 µm, 2.1 x 50 mm. Gradient conditions: 5% to 95% CH3CN in H2O (0.1% formic acid) over 1 min, hold at 95% CH3CN for 0.1 min, 0.5 mL/min, 40 ºC. Optical specific rotations were obtained using JASCO P-2000 Digital Polarimeter equipped with Tungsten-Halogen lamp (WI), 589 nm wavelength, photomultiplier tube (1P28-01) detector and CG2-100 Cylindrical glass cell, 2.5ø x 100 mm. For compounds that were purified on a Gilson preparative reversed-phase HPLC, the system comprised of a 333 aqueous pump with solvent-selection valve, 334 organic pump, GX271 or GX-281 liquid hander, two column switching valves, and a 155 UV detector. UV wavelength for fraction collection S3 was user-defined, with absorbance at 254 nm always monitored. Method 1: Phenomenex Axia-packed Luna C18, 30 x 50 mm, 5 µm column. Mobile phase: CH3CN in H2O (0.1% TFA). Gradient conditions: 0.75 min equilibration, followed by user defined gradient (starting organic percentage, ending organic percentage, duration), hold at 95% CH3CN in H2O (0.1% TFA) for 1 min, 50 mL/min, 23 ºC. Method 2: Phenomenex Axiapacked Gemini C18, 50 x 250 mm, 10 um column. Mobile phase: CH3CN in H2O (0.1% TFA). Gradient conditions: 7 min equilibration, followed by user defined gradient (starting organic percentage, ending organic percentage, duration), hold at 95% CH3CN in H2O (0.1% TFA) for 7 min, 120 mL/min, 23 ºC. Chiral separation was performed on a Thar (Waters) Investigator SFC Column: Chiral Technologies CHIRALPAK IF, 4.6 x 250 mm, 5 µm column. Gradient conditions: 20% to 50% IPA in CO2 over 7 min, hold at 50% CO2 for 1 min. Flow rate: 3.5 mL/min. Column temperature: 40 ºC. System backpressure: 100 bar. Solvents for extraction, washing and chromatography were HPLC grade. All reagents were purchased from Aldrich Chemical Co. and were used without purification. All compounds were >95% pure by LCMS (214 nm, 254 nM and ELSD) and final compounds were checked for confirmation of purity via ^{1}H and ^{13}CNMR

Preparation of VU6066098

6-Bromo-4-chloroisoindolin-1-one (Intermediate B).

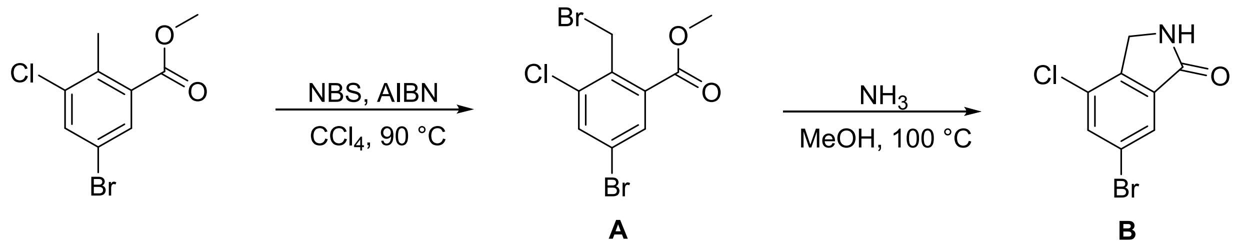

Methyl 5-bromo-2-(bromomethyl)-3-chlorobenzoate (Intermediate A). To a solution of methyl 5-bromo-3-chloro-2-methylbenzoate (3.0 g, 11.4 mmol) in carbon tetrachloride (53 mL) was added N-bromosuccinimide (2.21 g, 1.1 mmol) and AIBN (195 mg, 0.1 mmol). The reaction mixture was heated to 90 °C. After 3 h, the reaction mixture was washed with water (20 mL) and separated. The aqueous layer was extracted with DCM (2 x 20 mL) and the combined organic layers were washed with brine (100 mL). The organic layer was passed through a hydrophobic phase separator and concentrated *in vacuo* to give a crude residue. The residue was purified via normal phase column chromatography (0-100% hexanes/EtOAc) to provide **Intermediate A** (3.33 g, 85% yield). ^{1}H NMR (400 MHz, DMSO-*d6*) δ 8.11 (d, *J* = 2.1 Hz, 1H), 7.99 (d, *J* = 2.1 Hz, 1H), 4.96 (s, 2H), 3.89 (s, 3H); ES-MS $[M+1]^+$: 343.8.

6-Bromo-4-chloroisoindolin-1-one (Intermediate B). Methyl 5-bromo-2-(bromomethyl)-3-chlorobenzoate (3.33 g, 9.73 mmol) was dissolved in a 7N solution of ammonia in methanol (36.1 mL). The reaction mixture stirred in a Teflon® screwcap flask and was heated to 100 °C. After 3 h, the cap was carefully removed and the solvent was then evaporated *in vacuo* and the crude residue was purified by normal phase column chromatography (0-10% EtOAc/MeOH) to give the title compound (1.99 g, 83% yield). ^{1}H NMR (400 MHz, DMSO-*d6*) δ 8.95 (s, 1H), 7.97 (d, *J* = 1.6 Hz, 1H), 7.79 (d, *J* = 1.6 Hz, 1H), 4.36 (s, 2H); ES-MS $[M+1]^+$: 245.9 and 247.9.

4-Chloro-6-(2,4-difluorophenyl)isoindolin-1-one (Intermediate C).

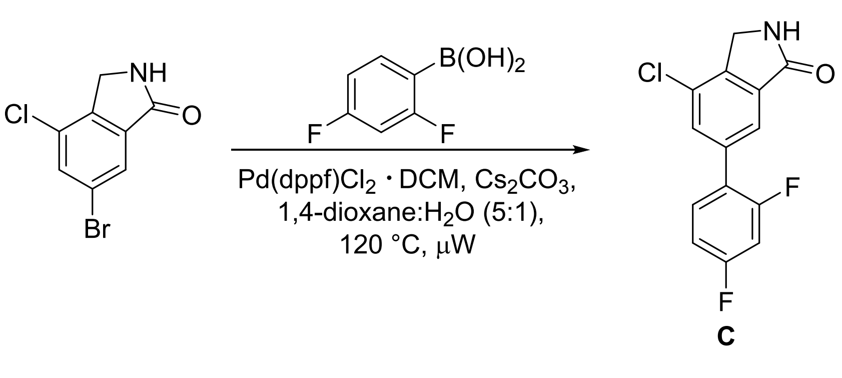

Equally separated in 2 x 20 mL microwave vials, to a mixture of 6-bromo-4-chloroisoindolin-1-one (1.64 g, 6.65 mmol), Pd(dppf)Cl_2 • DCM (544 mg, 0.67 mmol), 2,4 difluorophenyl boronic acid (1.16 g, 7.32 mmol), and Cs_2CO_3 (2.18 g, 6.65 mmol) was added degassed 1,4-dioxane (24.3 mL) and water (4.9 mL). The reaction atmosphere was evacuated and purged with nitrogen (3x) and the sealed reaction vessel underwent microwave irradiation at 120 °C for 15 min. The reaction mixture was concentrated. To the crude residue was added DCM (40 mL) and a precipitate was formed. The reaction mixture was filtered and the precipitate was isolated to afford the title compound (1.86 g, 99% yield). ES-MS $[M+1]^+$: 280.1, ^{1}H NMR (400 MHz, DMSO-*d6*) δ 8.91 (s, 1H), 7.86 – 7.82 (m, 1H), 7.76 – 7.73 (m, 1H), 7.73 – 7.68 (m, 1H), 7.43 (ddd, *J* = 11.5, 9.3, 2.6 Hz, 1H), 7.23 (td, *J* = 8.2, 2.4 Hz, 1H), 4.44 (s, 2H).

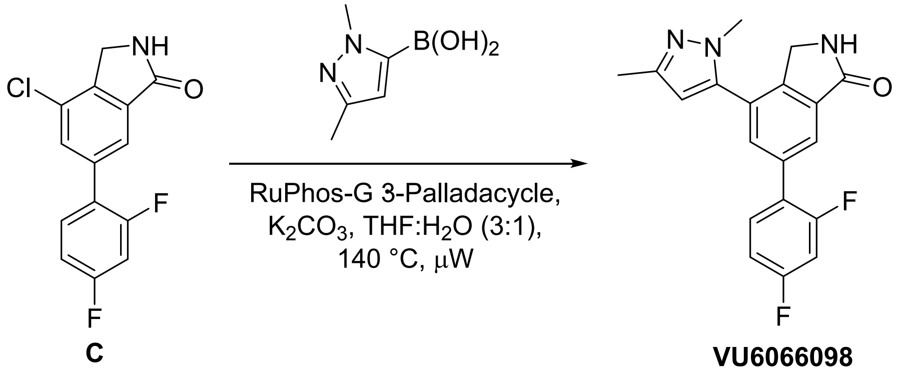

6-(2,4-difluorophenyl)-4-(1,3-dimethyl-1H-pyrazol-5-yl)isoindolin-1-one (VU6066098). Equally separated between 5 x 20 mL microwave vial was added potassium carbonate (2.8 g, 19.95 mmol), 1,3-dimethylpyrazole-5-boronic acid (2.33 g, 16.6 mmol), RuPhos-G3-Palladacycle (CAS# 1445085-77-7) (0.56 g, 0.67 mmol) and 4-Chloro-6-(2,4-difluorophenyl)isoindolin-1-one (1.86 g, 6.65 mmol). The reaction was evacuated and purged with nitrogen (3x). To each vial was added THF:water (14 mL; 3:1) and the vial was subjected to microwave irradiation at 140 °C for 15 min. Each reaction mixture was diluted with DCM (25 mL) at rt and filtered through a hydrophobic phase separator. The collected organic layers were concentrated and purified via reverse phase column chromatography (10-55% acetonitrile/ 0.05% aqueous NH_4OH) to provide desired product (1.77g, 78% yield) as a white solid. ES-MS $[M+1]^+$: 340.1; ^{1}H NMR (400 MHz, DMSO-d_6) δ 8.81 (s, 1H), 7.82 (t, *J* = 1.6 Hz, 1H), 7.79 (t, *J* = 1.5 Hz, 1H), 7.74 (td, *J* = 8.9, 6.6 Hz, 1H), 7.43 (ddd, *J* = 11.6, 9.3, 2.6 Hz, 1H), 7.27 – 7.20 (m, 1H), 6.42 (s, 1H), 4.42 (s, 2H), 3.75 (s, 3H), 2.20 (s, 3H); ^{13}C NMR (101 MHz, DMSO-d_6) δ 169.2, 162.1 (dd, *J* = 247.6, 12.4 Hz), 159.2 (dd, *J* = 248.9, 12.4 Hz), 146.3, 142.5, 138.9, 134.4, 134.1, 132.3 (dd, *J* = 9.8, 4.3 Hz), 132.0 (d, *J* = 2.5 Hz), 126.7, 123.8 (dd, *J* = 13.2, 3.7 Hz), 122.9 (d, *J* = 3.1 Hz), 112.3 (dd, *J* = 21.1, 3.7 Hz), 106.0, 104.7 (t, *J* = 26.4 Hz), 44.7, 36.9, 13.3; HR-MS (Q-TOF, ES+) calc'd for C19H15F2N3O, 340.1256; found, 340.1260.

NMR Spectra

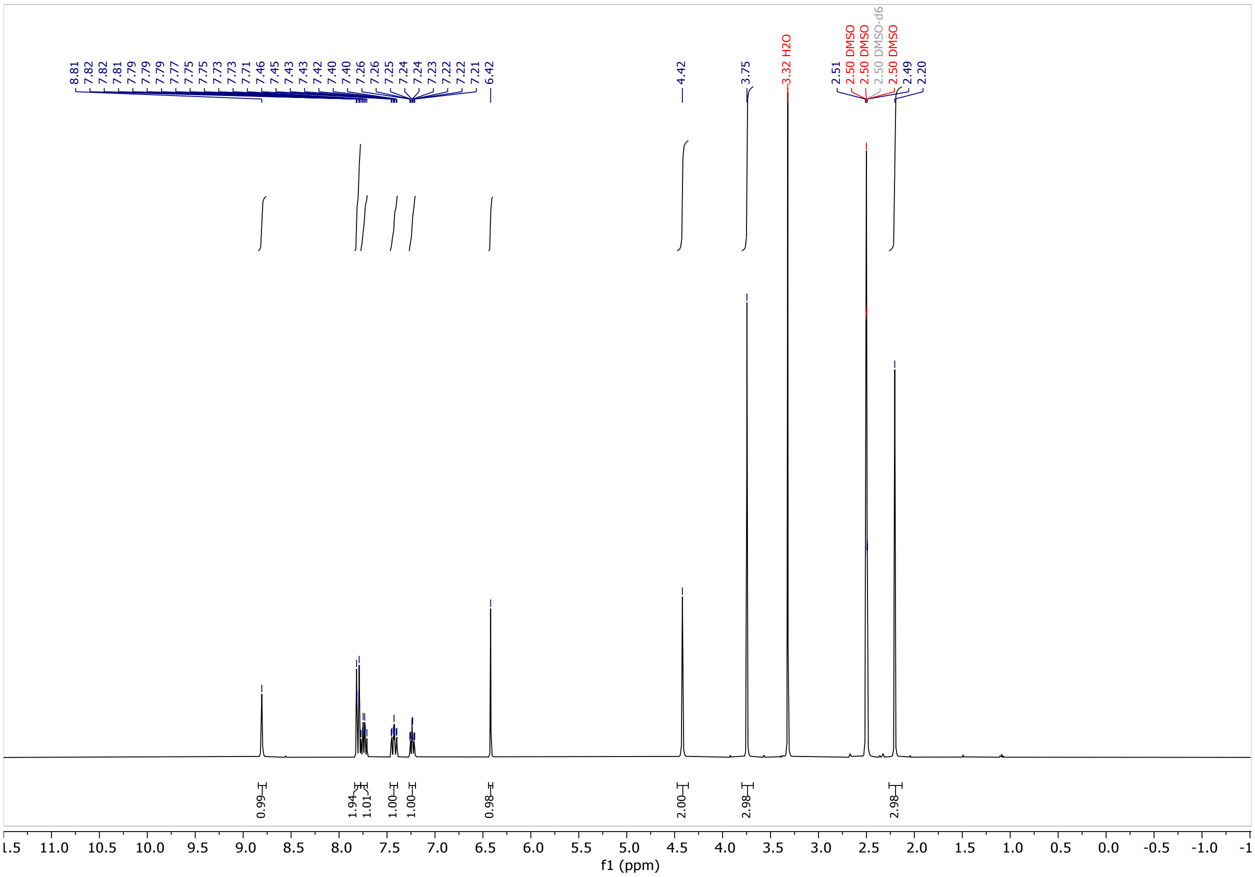

^{1}H NMR (400 MHz, DMSO-d_6) δ 8.81 (s, 1H), 7.82 (t, J = 1.6 Hz, 1H), 7.79 (t, J = 1.5 Hz, 1H), 7.74 (td, J = 8.9, 6.6 Hz, 1H), 7.43 (ddd, J = 11.6, 9.3, 2.6 Hz, 1H), 7.27 – 7.20 (m, 1H), 6.42 (s, 1H), 4.42 (s, 2H), 3.75 (s, 3H), 2.20 (s, 3H).

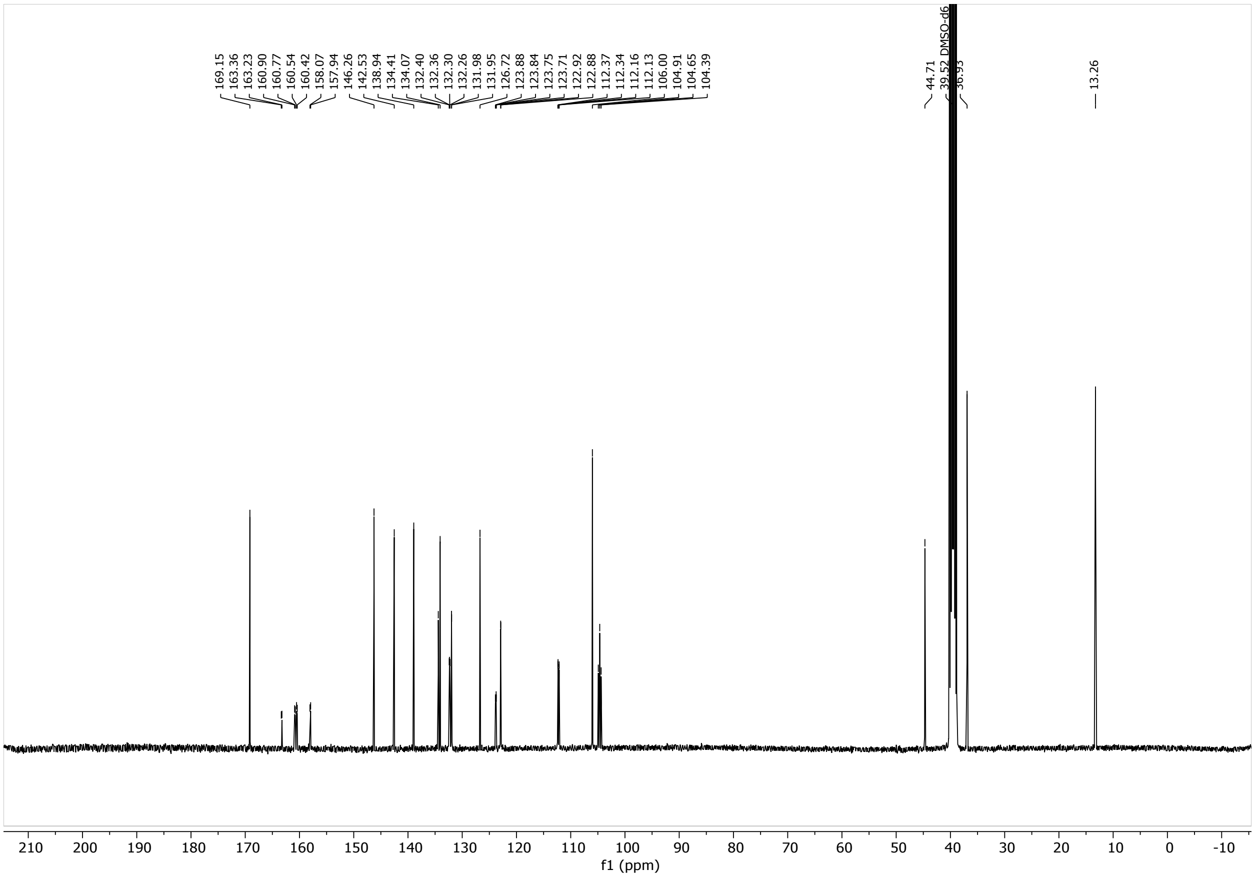

^{13}C NMR (101 MHz, DMSO-d_6) δ 169.2, 162.1 (dd, J = 247.6, 12.4 Hz), 159.2 (dd, J = 248.9, 12.4 Hz), 146.3, 142.5, 138.9, 134.4, 134.1, 132.3 (dd, J = 9.8, 4.3 Hz), 132.0 (d, J = 2.5 Hz), 126.7, 123.8 (dd, J = 13.2, 3.7 Hz), 122.9 (d, J = 3.1 Hz), 112.3 (dd, J = 21.1, 3.7 Hz), 106.0, 104.7 (t, J = 26.4 Hz), 44.7, 36.9, 13.3.

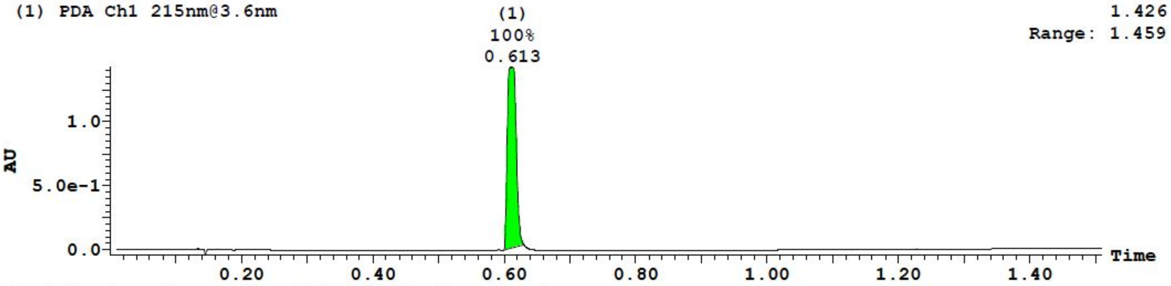

Peak Number	Time	Area %Total	BPM	Mass Found
1	0.613	100.00	340	339.0000

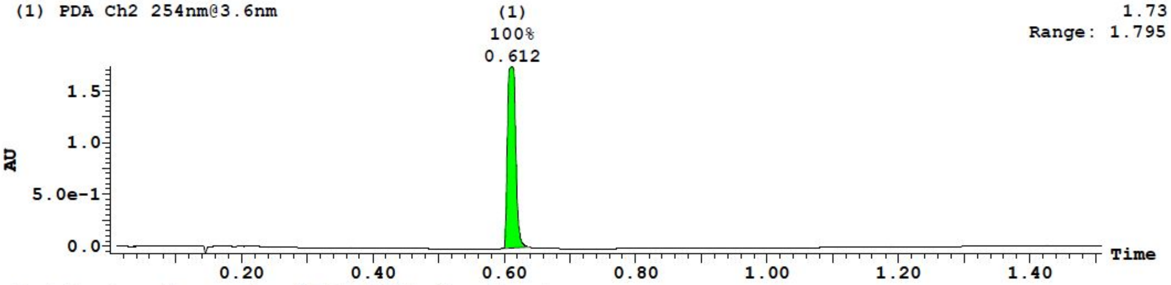

Peak Number	Time	Area %Total	BPM	Mass Found
1	0.612	100.00	340	339.0000

ES-MS $[M+1]^+$: 340.1

Eurofin Cerep Saftey Screen44.

7.1. *In Vitro* Pharmacology: Binding Assays

7.1.1. Test Compound Results

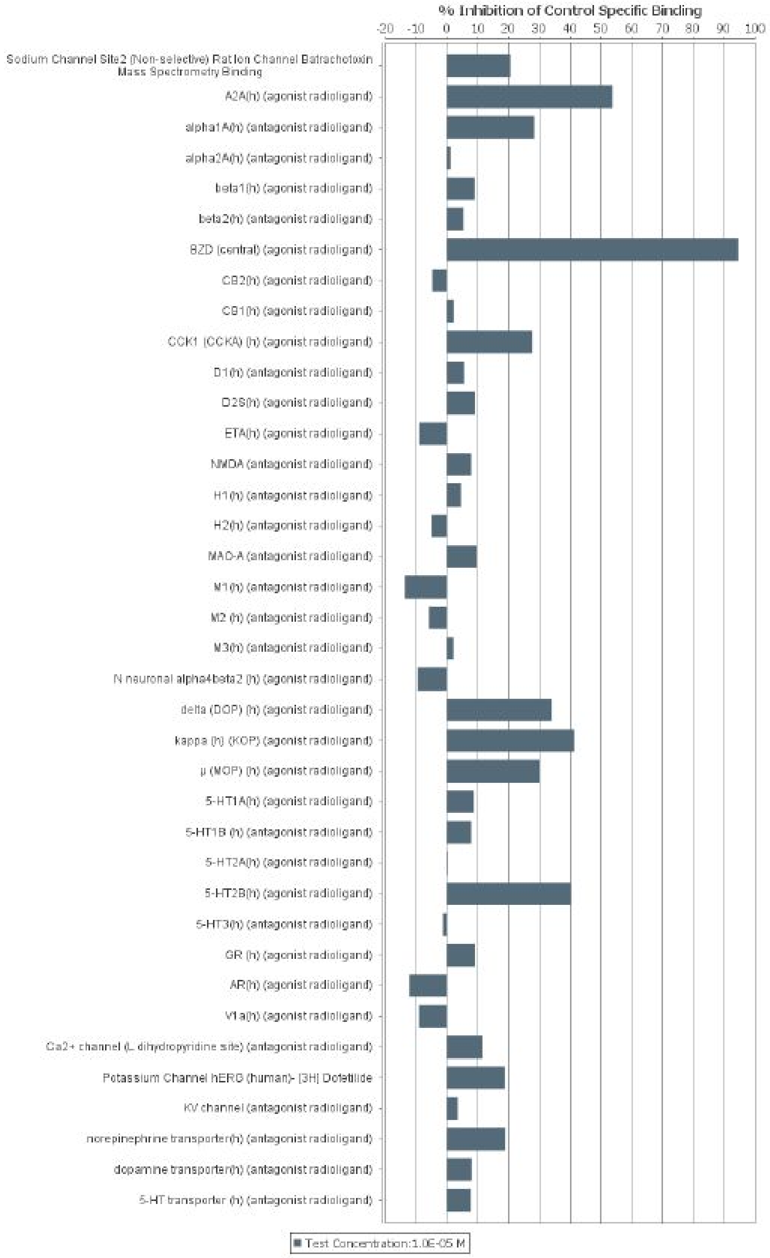

Figure 1. Histogram for VU6066098-05

PO PK of VU6066098-02 in Rat

Project	mGluR2_NAM	
Study	PO PK	
Animals	Rat	SD, male (n=2)
Vehicle	(PO) 10% Tween 80	
Dose	(PO) 10 mg/kg; 100 mg/kg	
Matrix	EDTA plasma	
Analyte	VU6066098-02	
LLOQ	1.0 ng/mL	

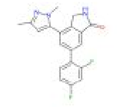

VU6066098-02

0.2 mg/kg IV VU6066098-02				
Dose (mg/kg)	Time (hr)	Concentration (ng/mL) 1	2	Ave
0.20	0.0333	167		167
	0.117	134		134
	0.25	124		124
	0.5	84.8		84.8
	1	70.0		70.0
	2	31.8		31.8
	4	4.93		4.93
	7	1.64		1.64
	24	BLQ		BLQ

	PK Parameter		1	2	Ave
IV PK Cassette 0.2 mpk	$t_{1/2 \text{(term)}}$	hr	1.23		1.23
	MRT	hr	1.56		1.56
	Cl obs	mL/min/kg	23.9		23.9
	Vss	L/kg	2.24		2.24
	AUC (IV)	hr*ng/mL	139		139
10 mpk PO PK	Cmax	ng/mL	2694	2949	2822
	Tmax	hr	2.00	1.00	1.50
	AUC (PO)	hr*ng/mL	19297	12432	15865
	F	%	277	179	228
100 mpk PO PK	Cmax	ng/mL	9374	8615	8995
	Tmax	hr	2.00	1.00	1.50
	AUC (PO)	hr*ng/mL	180118	120337	150228
	F	%	259	173	216

Oral F calculated using IV PK Cassette data C452

10 mg/kg PO VU6066098-02				
Dose (mg/kg)	Time (hr)	Concentration (ng/mL) 1	2	Ave
10	0.25	739	2214	1476
	0.5	1446	2643	2044
	1	2458	2949	2704
	2	2694	2394	2544
	4	2014	1489	1752
	7	762	134	448
	24	2.69	1.89	2.29
	48	1.25	1.39	1.32

Time points in yellow not used in PK analysis to achieve good correlation.

100 mg/kg PO VU6066098-02				
Dose (mg/kg)	Time (hr)	Concentration (ng/mL) 1	2*	Ave
100	0.25	3241	2952	3096
	0.5	5923	6415	6169
	1	7531	8615	8073
	2	9374	8410	8892
	4	7196	6890	7043
	7	5485	3649	4567
	24	237	4476	2356
	48	502	21.7	262

Time points in yellow not used in PK analysis to achieve good correlation.
*PO PK data of rat 2 has a R2 correlation of 0.80.

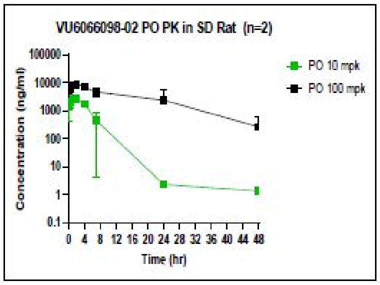

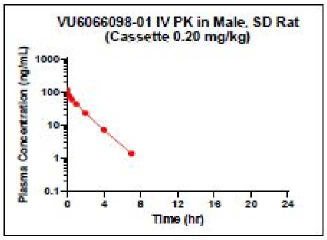

Supplemental Figure 1. Rat IV/PO PK with NAM 7.

VU6066098-02 in PBL

Project	mGlu2 NAM	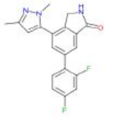
Study	PBL	
Animals	S-D rat (Male, n = 3)	
Vehicle	10% Tween 80	
Dose	10 mg/kg	
Time point	30, 60, 120, 240, 360, 720, and 1440 min after dosing	
Matrix	Plasma (EDTA), Whole Brain	
Analyte	VU6066098-02	
LLOQ	Plasma 1.0 ng/ml; Brain 0.5 ng/mL	VU6066098-02

VU6066098-02							
Time (hr)	Animal	Plasma (ng/mL)	Ave	Brain (ng/g)	Ave	Brain/Plasma Ratio	Ave
0.5	A4	3350	3357	3133	3277.2	0.94	0.96
	A5	3909		4237		1.08	
	A6	2812		2461		0.88	
1	A7	4257	4797	4865	4992.5	1.14	1.05
	A8	5018		5177		1.03	
	A9	5116		4935		0.96	
2	A10	4028	4374	3478	3904.9	0.86	0.89
	A11	4354		3936		0.90	
	A12	4739		4300		0.91	
4	C1	2507	2220	2348	1858.2	0.94	0.83
	C2	2063		1594		0.77	
	C3	2089		1632		0.78	
6	C4	1474	1467	1077	1207.7	0.73	0.82
	C5	2066		1795		0.87	
	C6	859		751		0.87	
12	C7	4.66	7.91	6.63	8.09	1.42	1.16
	C8	16.6		14.8		0.89	
	C9	2.44		2.87		1.18	
24	C10	0.17	0.16	1.34	0.78	7.90	4.65
	C11	0.21		0.68		3.24	
	C12	0.11		0.31		2.81	

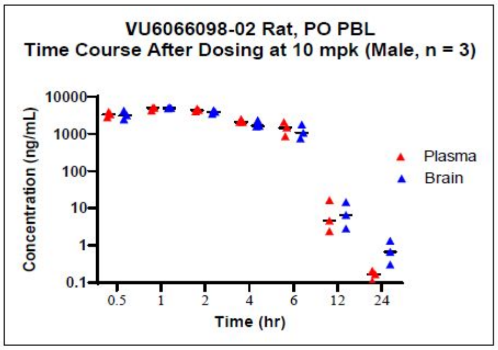

VU6066098-02 RAT_PO PBL								
VU#	Time (hr)	Plasma (ng/mL)	Plasma Total (μM)	Plasma Free (nM)	Brain (ng/mL)	Brain Total (μM)	Brain Free (nM)	Fold EC50
VU6066098-02	0.5	3357	9.90	695	3277	9.66	130	0.93
	1	4797	14.1	993	4992	14.7	199	1.42
	2	4374	12.9	905	3905	11.5	155	1.11
	4	2220	6.55	460	1858	5.48	74.0	0.53
	6	1467	4.32	304	1208	3.56	48.1	0.34
	12	7.91	0.02	1.64	8.09	0.02	0.32	0.00
	24	0.16	0.00	0.03	0.78	0.00	0.03	0.00

VU#	MW	Plasma fu	Brain fu	IC50 (nM)
VU6066098-02	339.1183	0.0702	0.0135	140

Supplemental Figure 2. Rat PO PBL timecourse with NAM 7.
